# Exploring diarylheptanoid derivatives to target LIMK1 as potential agents against colorectal cancer

**DOI:** 10.1080/14756366.2025.2583826

**Published:** 2025-11-17

**Authors:** Liang-Chieh Chen, Tung-Cheng Chang, Hui-Ju Tseng, Jung-Chun Chu, Yun-Yi Huang, Hao-Yuan Peng, Yi-Chen Kuo, Yi-Wen Wu, Tony Eight Lin, Shih-Chung Yen, Kai-Cheng Hsu, Wei-Jan Huang, Shiow-Lin Pan

**Affiliations:** ^a^ School of Medicine, College of Medicine, National Sun Yat-sen University, Kaohsiung, Taiwan; ^b^ Innovation Center for Drug Development and Optimization, National Sun Yat-sen University, Kaohsiung, Taiwan; ^c^ Institute of BioPharmaceutical Sciences, College of Medicine, National Sun Yat-sen University, Kaohsiung, Taiwan; ^d^ Institute of Precision Medicine, College of Medicine, National Sun Yat-sen University, Kaohsiung, Taiwan; ^e^ Division of Colorectal Surgery, Department of Surgery, Shuang Ho Hospital, Taipei Medical University, Taipei, Taiwan; ^f^ Division of General Surgery, Department of Surgery, School of Medicine, College of Medicine, Taipei Medical University, Taipei, Taiwan; ^g^ School of Pharmacy, College of Pharmacy, Kaohsiung Medical University, Kaohsiung, Taiwan; ^h^ Ph.D. Program in Drug Discovery and Development Industry, College of Pharmacy, Taipei Medical University, Taipei, Taiwan; ^i^ Graduate Institute of Pharmacognosy, College of Pharmacy, Taipei Medical University, Taipei, Taiwan; ^j^ Graduate Institute of Cancer Biology and Drug Discovery, College of Medical Science and Technology, Taipei Medical University, Taipei, Taiwan; ^k^ Ph.D. Program for Cancer Molecular Biology and Drug Discovery, College of Medical Science and Technology, Taipei Medical University, Taipei, Taiwan; ^l^ Warshel Institute for Computational Biology, School of Medicine, The Chinese University of Hong Kong (Shenzhen), Shenzhen, China; ^m^ TMU Research Center of Cancer Translational Medicine, Taipei Medical University, Taipei, Taiwan; ^n^ School of Pharmacy, College of Pharmacy, Taipei Medical University, Taipei, Taiwan

**Keywords:** LIMK1 inhibitor, diarylheptanoid, colorectal cancer, catechol, kinase inhibitor

## Abstract

LIMK1 has been demonstrated to be highly correlated with the progression and overall survival rates of colorectal cancer (CRC) patients. In this study, a series of diarylheptanoid scaffold derivatives were intentionally designed and synthesised to evaluate their potential as LIMK1 inhibitors. Among these compounds, compounds **13a** and **XV** exhibited LIMK1 inhibitory activity with IC_50_ values of 0.94 and 0.57 µM, respectively. We also disclosed the structure–activity relationship of the resulting compounds that exhibited LIMK1 inhibition. Catechol-containing diarylheptanoid was identified as a promising scaffold for LIMK1 inhibitors. Notably, compound **13a** demonstrated selectivity in inhibiting the tyrosine kinase-like family and exhibited potent inhibition of CRC cells. Moreover, compound **13a** induced an increase in the S phase and a decrease in the G0/G1 phase in a dose-dependent manner, indicating apoptosis induction. These findings establish compound **13a** as a lead compound for the further development of anti-CRC agents.

## GRAPHICAL ABSTRACT

**Figure:**
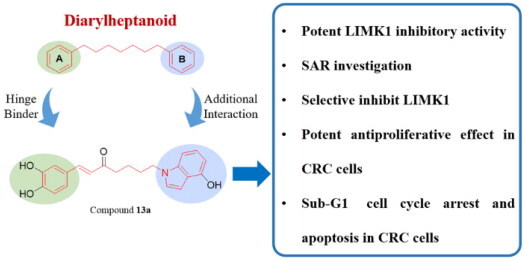

## Introduction

The LIM kinase family consists of two members, LIMK1 and LIMK2, collectively known as LIMK^1^. Both kinases have two N-terminal LIM domains, a PDZ domain connected to proline/serine-rich regions, and a C-terminal kinase domain^1^^,^^2^. The LIM domains are protein-binding motifs that interact with substrates, such as cofilin1, cofilin2, and actin-depolymerising factor (ADF)^1^^,^^3^. ADF/cofilin family can bind to actin and regulate actin filament dynamics, which is essential for cell migration, morphogenesis, and cell division^4^. Active cofilin/ADF can convert filamentous actin (F-actin) into monomeric globular actin (G-actin), promoting actin turnover and modulating cell motility. In contrast, inactivated cofilin/ADF loses its function, leading to the accumulation of actin polymers, stabilising actin filaments, and suppressing actin turnover^4^^,^^5^. LIMK can phosphorylate cofilin/ADF on serine 3, leading to cofilin inactivation and inhibition of its function^6^^,^^7^. Through regulating cofilin activity, LIMK can modulate actin cytoskeletal dynamics during cell migration^8^.

LIMK1 is overexpressed and essential in various types of cancer^9–12^. Studies have indicated that overexpressed LIMK1 can boost the invasion, metastatic ability, and progression of cancer cells^12–15^. Protein kinase C ζ (PKCζ) knockdown is linked to reduced phosphorylation of LIMK1 and cofilin, leading to the inhibition of migration of glioblastoma cells and macrophages^16^^,^^17^. Knockdown or inhibition of LIMK1 inhibits the proliferation of lung cancer, gastric cancer, colorectal cancer (CRC), and acute myeloid leukaemia (AML) cells, as well as the invasion motility of glioblastoma^11^^,^^12^^,^^18–20^. Furthermore, LIMK1 knockdown did not exhibit significant toxicity in mouse models of gastric cancer and glioblastoma, suggesting that LIMK1 could be a potential target without serious health concerns *in vivo*^12^^,^^19^.

The expression of LIMK1 is elevated in all stages of CRC^21^,^22^ which is one of the leading causes of cancer-related deaths globally. Analysis of 143 CRC samples revealed markedly elevated cytoplasmic expression of LIMK1 (93.7%), LIMK2 (89.5%), and cofilin (86.7%), while nuclear expression was detected in only 54.5%, compared with adjacent adenomas and non-neoplastic epithelium^23^. These findings suggest that LIMK1, LIMK2, and cofilin are predominantly activated in the cytoplasm of CRC cells, supporting their role in cytoskeletal remodelling and tumour progression. In clinical cohorts, CRC patients with elevated LIMK1 levels exhibit reduced overall survival and heightened lymph node metastasis^22^. Approximately 20% of CRC patients experience metastasis, and 40% have recurrence after treatment^22^^,^^24^^,^^25^. Metastatic CRC is incurable for most patients and has a less than 20% 5-year survival rate^24^, highlighting the unmet medical need for CRC treatment. Unlike the decreased role of LIMK2 in CRC initiation, LIMK1 is overexpressed and drives the invasion and migration of CRC cells^9^^,^^21^. Elevated LIMK1 expression is significantly associated with adverse clinicopathological features, including lymphatic invasion and advanced pathological stage^21^. Numerous studies have demonstrated that LIMK1 is highly correlated with the progression and overall survival rates of CRC patients^15^^,^^20–22^. Clinical and transcriptomic analyses revealed that LIMK1 is highly expressed at both mRNA and protein levels in CRC^21^, with expression correlated to lymphatic invasion, advanced pathological stage, poor prognosis, and immune cell infiltration (CD4^+^ T cells, macrophages, and dendritic cells)^21^. Together, these findings indicate that LIMK1 is a critical driver of CRC progression and a promising therapeutic target.

**Figure 1.**
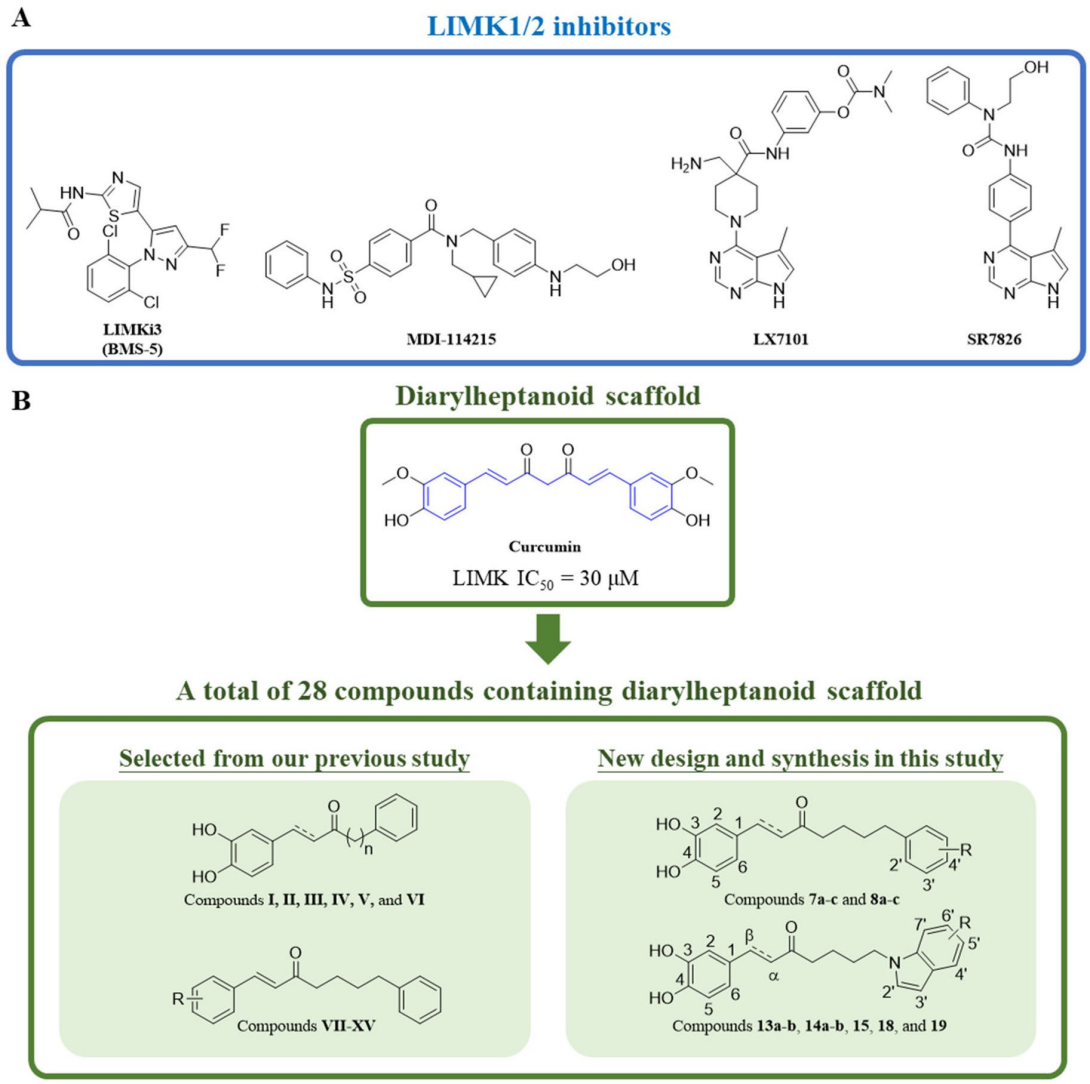
Representative chemical structures of (A) reported LIMK1/2 inhibitors and (B) the diarylheptanoid-based compound evaluated in this study.

Recent advances have clarified the landscape of small-molecule LIMK inhibitors. The thiazole derivative LIMKi3 (BMS-5) (Figure 1(A)), originally developed by Bristol-Myers Squibb, is a highly potent dual LIMK1 and LIMK2 inhibitor (IC_50_ = 6 and 33 nM, respectively) that effectively suppresses cofilin phosphorylation and demonstrates efficacy in cancer^26^ and Fragile X Syndrome^27^, but its further development has been hindered by poor kinase selectivity^26^^,^^28^^,^^29^. More recently, MDI-114215 (Figure 1(A)) has emerged as a selective and stable LIMK1/2 inhibitor that blocks both nonphosphorylated and PAK1-phosphorylated forms, suppresses cofilin phosphorylation, and rescues synaptic deficits in Fragile X Syndrome models and patient-derived neurons^30^. The pyrrolopyrimidines LX7101 (developed by Lexicon Pharmaceuticals) and SR7826 (Figure 1(A)) are potent LIMK inhibitors, with LX7101 advancing to clinical trials for glaucoma and SR7826 demonstrating neuroprotective effects in Fragile X and Alzheimer’s models^29–31^. Despite these promising activities, both compounds remain unselective LIMK1/2 inhibitors, limiting their value as tool molecules^29^^,^^31^. Together, these findings underscore the therapeutic promise of LIMK inhibition while highlighting the critical need for LIMK1/2 inhibitors to serve as reliable tool compounds and potential drug candidates.

Curcumin, an extensively researched secondary plant metabolite found in turmeric, exhibits significant anti-tumour effects in CRC or other cell lines, including cytotoxic effects, inhibited colony formation, and decreased cell motility^32–36^. Inhibition of cell motility by curcumin is mediated by reducing levels of active cofilin^32^. In a separate study, curcumin shows impressive LIMK inhibition with an IC_50_ value of 30 μM^37^. The main scaffold of curcumin is a diarylheptanoid that consists of 1,7-diphenylheptane and displays a wide variety of bioactivity^38^^,^^39^. The linear structure and multiple modified structural positions of diarylheptanoid provide abundant information for exploring the structure–activity relationship (SAR) study. Furthermore, the above study may connect and unveil the potential of diarylheptanoid scaffold derivatives for CRC and present promising opportunities for further research and applications. However, curcumin adopts a relatively rigid and extended conformation, which may restrict its ability to access critical binding residues. In contrast, the diarylheptanoid scaffold provides greater conformational flexibility, which can facilitate more favourable interactions with the target protein.

In this study, we designed and synthesised a series of diarylheptanoid scaffold derivatives and assessed their ability to inhibit LIMK1 (Figure 1(B)). 28 diarylheptanoid scaffold derivatives were assessed for their ability to inhibit LIMK1. Among them, 15 compounds were obtained from previous studies^37^. Besides that, we synthesised 13 compounds by adding a hydroxy group, which potentially forms additional interactions with LIMK1. To better understand the binding mechanism between LIMK1 and the compounds, we conducted docking and interaction analyses. Among the synthesised diarylheptanoids, the most potent compound was selected to assess its selectivity for other kinases. Additionally, its biological activity was evaluated through anti-proliferative assays and cell cycle analysis. Overall, these findings highlight that structurally modified diarylheptanoids can enhance LIMK1 inhibitory activity, providing additional options for optimising novel LIMK1 inhibitors.

## Materials and methods

### General procedures

Nuclear magnetic resonance spectra (^1^H and ^13^C NMR) were obtained using Bruker Fourier 300, Avance DRX 500, and AVIII 500 spectrometers. The chemical shifts were reported in parts per million (ppm, *δ*) with an internal standard, tetramethylsilane (TMS). The mass spectra were measured on THERMO Q Exactive Plus and Finnigan LCQ mass spectrometry. The melting points were determined on an FARGO MP-2D digital melting point apparatus. The purity of final compounds was determined using HPLC with a C18-column (150 mm × 4.6 mm, Ascentis) and an L-2130 pump (Hitachi, Ibaraki, Japan). Column chromatography was performed using silica gel (70–230 mesh, Merck, Darmstadt, Germany). The thin-layer chromatography analysis was performed using silica gel plates (KG60-F254, Merck). Unless otherwise mentioned, all chemicals were used without any purification, and all reactions were carried out under an atmosphere of dry nitrogen.

### (E)-Ethyl 2-benzyloxycinnamate (2a)

To a solution of NaH (2.26 g, 94.16 mmol) in dry tetrahydrofuran (THF) (50 ml) was added triethyl phosphonoacetate (18.7 ml, 94.25 mmol) in an ice bath. After stirring under N_2_ for 1 h, the resultant was added a solution of 2-benzyloxybenzaldehyde **1a** (10.0 g, 47.12 mmol) in dry THF and stirred for an additional 1 h at RT. The reaction mixture was diluted with distilled H_2_O and neutralised with 1 N HCl_(aq)_ to pH = 7. The mixture was extracted with EtOAc (3 × 50 ml). The combined organic layer was dried over anhydrous MgSO_4_, filtered, and the solvent was concentrated *in vacuo*. The residue was purified over a silica gel column eluted with 10% EtOAc in *n*-hexane to give compound **2a** (13.12 g, 99%) as a white solid. Melting point 50–52 °C. ^1^H NMR (Acetone-*d*_6_, 300 MHz): *δ* 8.06 (d, *J* = 16.1 Hz, 1H), 7.70 (dd, *J* = 7.7, 1.7 Hz, 1H), 7.52 (m, 2H), 7.38 (m, 4H), 7.18 (dd, *J* = 8.3, 0.8 Hz, 1H), 7.01 (ddd, *J* = 8.3, 7.7, 0.8 Hz, 1H), 6.58 (d, *J* = 16.1 Hz, 1H), 5.26 (s, 2H), 4.19 (q, *J* = 7.1 Hz, 2H), 1.27 (t, *J* = 7.1 Hz, 3H). ESI-MS *m/z*: 283.1 [M + H]^+^.

### (E)-ethyl 3-benzyloxycinnamate (2b)

Following the procedure as described for the preparation of compound **2a**, the reaction of NaH (2.26 g, 94.16 mmol) in THF (50 ml), triethyl phosphonoacetate (18.7 ml, 94.25 mmol), and 3-benzyloxybenzaldehyde (10.0 g, 47.11 mmol) in THF (50 ml) gave compound **2b** (11.00 g, 83%) as a white solid. Melting point 39–42 °C. ^1^H NMR (MeOH-*d*_4_, 300 MHz): *δ* 7.64 (d, *J* = 16.0 Hz, 1 H), 7.44 (m, 2H), 7.34 (m, 4H), 7.19 (m, 2H), 7.04 (ddd, *J* = 8.2, 2.5, 1.0 Hz, 1H), 6.49 (d, *J* = 16.0 Hz, 1H), 5.11 (s, 2H), 4.24 (q, *J* = 7.1 Hz, 2H), 1.32 (t, *J* = 7.1 Hz, 3H). ESI-MS *m/z*: 283.1 [M + H]^+^.

### (E)-ethyl 4-benzyloxycinnamate (2c)

Following the procedure as described for the preparation of compound **2a**, the reaction of NaH (2.26 g, 94.16 mmol) in THF (50 ml), triethyl phosphonoacetate (18.7 ml, 94.25 mmol), and 4-benzyloxybenzaldehyde (10.0 g, 47.11 mmol) in THF (50 ml) gave compound **2c** (13.30 g, 99%) as a white solid. Melting point 68–71 °C. ^1^H NMR (Acetone-*d*_6_, 300 MHz): *δ* 7.63 (m, 3H), 7.49 (m, 2H), 7.37 (m, 3H), 7.08 (ddd, *J* = 9.3, 2.6 Hz, 2H), 6.39 (d, *J* = 16.0 Hz, 1H), 5.19 (s, 2H), 4.19 (q, *J* = 7.1 Hz, 2H), 1.27 (t, *J* = 7.1 Hz, 3H). ESI-MS *m/z*: 283.1 [M + H]^+^.

### (E)-3-(2-(benzyloxy)phenyl)prop-2-en-1-ol (3a)

To a solution of compound **2a** (13.00 g, 46.04 mmol) in dry CH_2_Cl_2_ (100 ml) was added 1.2 M diisobutylaluminium hydride (DIBAL-H) in toluene (76.7 ml, 92.04 mmol) dropwise under −30 °C. The resultant was stirred under N_2_ for 3 h. The reaction mixture was quenched with distilled H_2_O at 0 °C, washed with CH_2_Cl_2_ (5 × 50 ml), and filtered through a celite pad. The filtrate was dried over anhydrous Na_2_SO_4_, filtered, and the solvent was concentrated *in vacuo*. The residue was purified over a silica gel column eluted with 20% EtOAc in *n*-hexane to give compound **3a** (10.96 g, 99%) as a white solid. Melting point 49–51 °C. ^1^H NMR (Acetone-*d*_6_, 300 MHz): *δ* 7.51 (m, 3H), 7.40 (m, 2H), 7.33 (m, 1H), 7.20 (ddd, *J* = 8.2, 7.2, 1.4 Hz, 1H), 7.06 (dd, *J* = 8.2, 1.0 Hz, 1H), 6.99 (ddd, *J* = 16.1, 1.7, 1.7 Hz, 1H), 6.92 (ddd, *J* = 7.4, 7.2, 1.0 Hz, 1H), 6.41 (dt, *J* = 16.1, 5.4 Hz, 1H), 5.17 (s, 2H), 4.23 (td, *J* = 5.7, 1.7 Hz, 2H), 3.79 (t, *J* = 5.7 Hz, 1H). ESI-MS *m/z*: 223.11 [M − OH]^+^.

### (E)-3-(3-benzyloxyphenyl)prop-2-en-1-ol (3b)

Following the procedure as described for the preparation of compound **3a**, the reaction of compound **2b** (7.40 g, 26.21 mmol) in dry CH_2_Cl_2_ (100 ml) was added 1.2 M DIBAL-H in toluene (43.7 ml, 52.44 mmol) to give compound **3b** (6.1 g, 97%) as a light yellow solid. Melting point 56–59 °C. ^1^H NMR (MeOD-*d*_4_, 300 MHz): *δ* 7.44 (m, 2H), 7.37 (m, 2H), 7.30 (m, 1H), 7.21 (dd, *J* = 7.9, 7.9 Hz, 1H), 7.01 (m, 2H), 6.86 (ddd, *J* = 8.2, 2.5, 0.7 Hz, 1H), 6.57 (m, 1H), 6.34 (dt, *J* = 15.9, 5.5 Hz, 1H), 6.34 (s, 2H), 4.21 (dd, *J* = 5.5, 1.5 Hz, 2H). ESI-MS *m/z*: 223.11 [M − OH]^+^.

### (E)-3-(4-benzyloxyphenyl)prop-2-en-1-ol (3c)

Following the procedure as described for the preparation of compound **3a**, the reaction of compound **2c** (13.30 g, 47.11 mmol) in dry CH_2_Cl_2_ (100 ml), and 1.2 M DIBAL-H in toluene (78.5 ml, 94.20 mmol) gave compound **3c** (10.87 g, 96%) as a white solid. Melting point 112–114 °C. ^1^H NMR (Acetone-*d*_6_, 300 MHz): *δ* 7.48 (m, 2H), 7.39 (m, 3H), 7.33 (m, 2H), 6.97 (d, *J* = 8.8 Hz, 2H), 6.54 (m, 1H), 6.26 (dt, *J* = 15.7, 5.4 Hz, 1H), 5.12 (s, 2H), 4.20 (ddd, *J* = 5.6, 5.5, 1.7 Hz, 2H), 3.75 (t, *J* = 5.6 Hz, 1H). ESI-MS *m/z*: 223.11 [M − OH]^+^.

### 2-Benzyloxycinnamaldehyde (4a)

To a solution of compound **3a** (10.86 g, 45.19 mmol) in toluene (100 ml) was added MnO_2_ (39.29 g, 451.92 mmol). The resultant was refluxed and stirred under N_2_ for 2 h. The reaction mixture was filtered through a celite pad, washed with toluene, and the filtrate was concentrated *in vacuo*. The residue was purified over a silica gel column eluted with 5% EtOAc in *n*-hexane to give compound **4a** (7.50 g, 69%) as a yellow solid. Melting point 63–65 °C. ^1^H NMR (Acetone-*d*_6_, 300 MHz): *δ* 9.68 (d, *J* = 7.7 Hz, 1 H), 7.96 (d, *J* = 16.1 Hz, 1 H), 7.74 (dd, *J* = 7.8, 1.8 Hz, 1H), 7.54 (m, 2H), 7.41 (m, 4H), 7.22 (dd, *J* = 8.4, 0.8 Hz, 1H), 7.05 (m, 1H), 6.82 (dd, *J* = 16.1, 7.8 Hz, 1H), 5.29 (s, 2H). ESI-MS *m/z*: 239.1 [M + H]^+^.

### 3-Benzyloxycinnamaldehyde (4b)

Following the procedure as described for the preparation of compound **4a**, the reaction of compound **3b** (9.0 g, 37.45 mmol) in toluene (100 ml) and MnO_2_ (32.56 g, 374.55 mmol) gave compound **4b** (7.19 g, 81%) as a yellow solid. Melting point 95–97 °C. ^1^H NMR (MeOH-*d*_4_, 300 MHz): *δ* 9.65 (d, *J* = 7.8 Hz, 1H), 7.63 (d, *J* = 15.9 Hz, 1H), 7.45 (m, 2H), 7.32 (m, 6H), 7.10 (ddd, *J* = 8.1, 2.5, 1.0 Hz, 1H), 6.75 (dd, *J* = 15.9, 7.8 Hz, 1H), 5.13 (s, 2H). ESI-MS *m/z*: 239.1 [M + H]^+^.

### 4-Benzyloxycinnamaldehyde (4c)

Following the procedure as described for the preparation of compound **4a**, the reaction of compound **3c** (10.86 g, 45.19 mmol) in toluene (100 ml) and MnO_2_ (39.19 g, 450.82 mmol) gave compound **4c** (7.32 g, 68%) as a white solid. Melting point 77–79 °C. ^1^H NMR (Acetone-*d*_6_, 300 MHz): *δ* 9.66 (d, *J* = 7.7 Hz, 1H), 7.70 (m, 2H), 7.62 (d, *J* = 15.9 Hz, 1 H), 7.50 (m, 2H), 7.38 (m, 3H), 7.12 (m, 2H), 6.66 (dd, *J* = 15.9, 7.7 Hz, 1H), 5.21 (s, 2H). ESI-MS *m/z*: 239.1 [M + H]^+^.

### *General procedure for the synthesis of compounds 5a*–*c*

To a solution of 1-(triphenylphosphoranylidene)-2-propanone (31.0 mmol) in dry toluene (50 ml) was added a solution of compounds **4a–c** (31.0 mmol) in dry toluene (50 ml). The resultant was refluxed and stirred under N_2_ for 12 h. The reaction mixture was concentrated *in vacuo*, extracted with EtOAc (200 ml), and washed with distilled H_2_O (3 × 100 ml). The organic layer was dried over anhydrous MgSO_4_, filtered, and the solvent was concentrated *in vacuo*. Compounds **5a–c** were obtained without further purification.

### 6-(2-Hydroxyphenyl)hexan-2-one (6a)

To a solution of compound **5a** (2.00 g, 7.19 mmol) in EtOH (15 ml) was added 10% Pd–C (400 mg). The resultant was stirred at room temperature under H_2_ atmosphere for 6 h. The reaction mixture was filtered through a celite pad, and the filtrate was concentrated *in vacuo*. The residue was purified over a silica gel column eluted with 10% EtOAc in *n*-hexane to give compound **6a** (1.38 g, 99%) as a brown solid. Melting point 35–37 °C. ^1^H NMR (Acetone-*d*_6_, 300 MHz): *δ* 8.10 (s, 1H), 7.08 (dd, *J* = 7.4, 1.8 Hz, 1H), 6.99 (ddd, *J* = 7.9, 7.5, 1.8 Hz, 1H), 6.80 (dd, *J* = 7.9, 1.2 Hz, 1H), 6.74 (ddd, *J* = 7.5, 7.4, 1.2 Hz, 1H), 2.61 (m, 2H), 2.47 (m, 2H), 2.06 (s, 3H), 1.58 (m, 4H). ESI-MS *m/z*: 193.1 [M + H]^+^.

### 6-(3-Hydroxyphenyl)hexan-2-one (6b)

Following the procedure as described for the preparation of compound **6a**, the reaction of compound **5b** (2.00 g, 7.19 mmol) in EtOH (15 ml), and 10% Pd–C (400 mg) gave compound **6b** (1.38 g, 99%) as a brown solid. Melting point 75–78 °C. ^1^H NMR (Acetone-*d*_6_, 300 MHz): *δ* 8.13 (s, 1H), 7.07 (m, 1H), 6.65 (m, 3H), 2.53 (m, 2H), 2.47 (m, 2H), 2.06 (s, 3H), 1.56 (m, 4H). ESI-MS *m/z*: 193.1 [M + H]^+^.

### 6-(4-Hydroxyphenyl)hexan-2-one (6c)

Following the procedure as described for the preparation of compound **6a**, the reaction of compound **5c** (2.0 g, 7.19 mmol) in EtOH (15 ml) and 10% Pd–C (400 mg) gave compound **6c** (1.38 g, 99%) as a white solid. Melting point 47–50 °C. ^1^H NMR (dimethyl sulfoxide-*d*_6_ (DMSO-*d*_6_), 300 MHz): δ 9.08 (s, 1H), 6.95 (d, *J* = 8.5 Hz, 2H), 6.65 (d, *J* = 8.5 Hz, 2H), 2.43 (m, 4H), 2.05 (s, 3H), 1.45 (m, 4H). ESI-MS *m/z*: 193.1 [M + H]^+^.

### 1-(3.4-Dihydroxyphenyl)-7-(2-hydroxyphenyl)hept-1-en-3-one (7a)

To a solution of compound **6a** (400 mg, 2.08 mmol) in THF (20 ml) was added pyrrolidine (171 μl, 2.08 mmol), acetic acid (119 μl, 2.08 mmol), and 3,4-dihydroxybenzaldehyde (287 mg, 2.08 mmol). The resultant was refluxed and stirred under N_2_ for 4 h. The reaction mixture was diluted with EtOAc (100 ml) and washed with distilled H_2_O (3 × 50 ml). The organic layer was dried over anhydrous Na_2_SO_4_, filtered, and the solvent was concentrated *in vacuo*. The residue was purified over a silica gel column eluted with 2.5% MeOH in CH_2_Cl_2_ to give compound **7a** (182 mg, 28%) as a brown solid. Melting point 158–162 °C. ^1^H NMR (Acetone-*d*_6_, 500 MHz): *δ* 8.48 (br, 1H), 8.19 (br, 2H), 7.47 (d, *J* = 16.1 Hz, 1H), 7.17 (d, *J* = 2.2 Hz, 1H), 7.10 (dd, *J* = 7.4, 1.6 Hz, 1H), 7.05 (dd, *J* = 8.2, 2.2 Hz, 1H), 6.99 (ddd, *J* = 7.9, 7.4, 1.6 Hz, 1H), 6.86 (d, *J* = 8.2 Hz, 1H), 6.81 (dd, *J* = 7.9, 1.1 Hz, 1H), 6.74 (ddd, *J* = 7.4, 1.1 Hz, 1H), 6.61 (d, *J* = 16.1 Hz, 1H), 2.66 (m, 4H), 1.66 (m, 4H). ^13^C NMR (Acetone-*d*_6_, 125 MHz): *δ* 200.1, 156.0, 148.8, 146.4, 143.1, 130.9, 129.6, 128.1, 127.7, 124.6, 122.8, 120.3, 116.5, 115.9, 115.3, 40.9, 30.6, 30.4, 25.1. HR-ESI-MS *m/z*: [M + H]^+^ calcd for C_19_H_21_O_4_ 313.1434, found 313.1434.

### 1-(3.4-Dihydroxyphenyl)-7-(3-hydroxyphenyl)hept-1-en-3-one (7b)

Following the procedure as described for the preparation of compound **7a**, the reaction of compound **6b** (150 mg, 0.78 mmol) in THF (10 ml), pyrrolidine (64 μl, 0.78 mmol), acetic acid (45 μl, 0.78 mmol), and 3,4-dihydroxybenzaldehyde (72 mg, 0.52 mmol) gave compound **7b** (46 mg, 28%) as a brown solid. Melting point 145–148 °C. ^1^H NMR (Acetone-*d*_6_, 500 MHz): *δ* 8.28 (br, 3H), 7.48 (d, *J* = 16.1 Hz, 1H), 7.17 (d, *J* = 2.1 Hz, 1H), 7.07 (m, 2H), 6.87 (d, *J* = 8.2 Hz, 1H), 6.64 (m, 4H), 2.68 (t, *J* = 7.0 Hz, 2H), 2.57 (t, *J* = 7.0 Hz, 2H), 1.65 (m, 4H). ^13^C NMR (Acetone-*d*_6_, 125 MHz): *δ* 199.9, 158.4, 148.8, 146.4, 144.9, 143.1, 130.1, 128.1, 124.6, 122.8, 120.5, 116.5, 116.2, 115.3, 113.6, 40.8, 36.4, 31.9, 24.9. HR-ESI-MS *m/z*: [M + H]^+^ calcd for C_19_H_21_O_4_ 313.1434, found 313.1434.

### 1-(3.4-Dihydroxyphenyl)-7-(4-hydroxyphenyl)hept-1-en-3-one (7c)

Following the procedure as described for the preparation of compound **7a**, the reaction of compound **6c** (500 mg, 2.60 mmol) in THF (20 ml), pyrrolidine (214 μl, 2.60 mmol), acetic acid (149 μl, 2.60 mmol), and 3,4-dihydroxybenzaldehyde (239 mg, 1.73 mmol) gave compound **7c** (159 mg, 29%) as a yellow solid. Melting point 148–151 °C. ^1^H NMR (Acetone-*d*_6_, 500 MHz): *δ* 8.21 (br, 3H), 7.47 (d, *J* = 16.2 Hz, 1H), 7.1 (d, *J* = 2.1 Hz, 1H), 7.05 (dd, *J* = 8.2, 2.1 Hz, 1H), 7.02 (d, *J* = 8.5 Hz, 2H), 6.87 (d, *J* = 8.2 Hz, 1H), 6.74 (d, *J* = 8.5 Hz, 2H), 6.60 (d, *J* = 16.2 Hz, 1H), 2.66 (t, *J* = 7.0 Hz, 2H), 2.55 (t, *J* = 7.2 Hz, 2H), 1.62 (m, 4H). ^13^C NMR (Acetone-*d*_6_, 125 MHz): *δ* 200.0, 156.3, 148.8, 146.4, 143.1, 134.0, 130.1, 128.0, 124.6, 122.8, 116.5, 116.0, 115.3, 40.8, 35.5, 32.3, 24.8. HR-ESI-MS *m/z*: [M + H]^+^ calcd for C_19_H_21_O_4_ 313.1434, found 313.1433.

### 1-(3.4-Dihydroxyphenyl)-7-(2-hydroxyphenyl)heptan-3-one (8a)

Following the procedure as described for the preparation of compound **6a**, the reaction of compound **7a** (182 mg, 0.58 mmol) in EtOH (10 ml) and 10% Pd–C (18 mg) gave compound **8a** (80 mg, 44%) as a yellow solid. Melting point 123–126 °C. ^1^H NMR (Acetone-*d*_6_, 500 MHz): *δ* 8.12 (s, 1H), 7.69 (s, 1H), 7.64 (s, 1H), 7.07 (dd, *J* = 7.5, 1.6 Hz, 1H), 6.99 (ddd, *J* = 7.9, 7.5, 1.6 Hz, 1H), 6.80 (dd, *J* = 7.9, 1.1 Hz, 1H), 6.74 (ddd, *J* = 7.5, 7.4, 1.1 Hz, 1H), 6.70 (d, *J* = 8.0 Hz, 1H), 6.68 (s, *J* = 2.1 Hz, 1H), 6.51 (dd, *J* = 8.0, 2.1 Hz, 1H), 2.68 (m, 4H), 2.60 (m, 2H), 2.45 (m, 2H), 1.57 (m, 4H). ^13^C NMR (Acetone-*d*_6_, 125 MHz): *δ* 210.0, 156.0, 145.9, 144.1, 134.2, 130.9, 129.5, 127.7, 120.4, 116.3, 116.1, 115.8, 45.1, 43.1, 30.7, 30.3, 29.9, 24.4. HR-ESI-MS *m/z*: [M + H]^+^ calcd for C_19_H_23_O_4_ 315.1591, found 315.1591.

### 1-(3.4-Dihydroxyphenyl)-7-(3-hydroxyphenyl)heptan-3-one (8b)

Following the procedure as described for the preparation of compound **6a**, the reaction of compound **7b** (80 mg, 0.26 mmol) in EtOH (5 ml) and 10% Pd–C (8 mg) to give compound **8b** (42 mg, 52%) as a brown solid. Melting point 98–101 °C. ^1^H NMR (Acetone-*d*_6_, 500 MHz): *δ* 8.12 (s, 1H), 7.67 (s, 1H), 7.63 (s, 1H), 7.07 (t, *J* = 7.8, Hz, 1H), 6.70 (d, *J* = 8.0 Hz, 1H), 6.65 (m, 4H), 6.51 (dd, *J* = 8.0, 2.1 Hz, 1H), 2.68 (m, 4H), 2.51 (m, 2H), 2.44 (m, 2H), 1.55 (m, 4H). ^13^C NMR (Acetone-*d*_6_, 125 MHz): *δ* 209.9, 158.3, 145.8, 144.9, 144.1, 134.2, 130.1, 120.5, 120.4, 116.3, 116.2, 116.1, 113.5, 45.0, 43.0, 36.3, 31.7, 29.9, 24.1. HR-ESI-MS *m/z*: [M + H]^+^ calcd for C_19_H_23_O_4_ 315.1591, found 315.1588.

### 1-(3.4-Dihydroxyphenyl)-7-(4-hydroxyphenyl)heptan-3-one (8c)

Following the procedure as described for the preparation of compound **6a**, the reaction of compound **7c** (100 mg, 0.32 mmol) in EtOH (5 ml) and 10% Pd–C (10 mg) to give compound **8c** (51 mg, 51%) as a brown solid. Melting point 90–93 °C. ^1^H NMR (Acetone-*d*_6_, 500 MHz): *δ* 8.08 (s, 1H), 7.71 (s, 1H), 7.67 (s, 1H), 6.99 (d, *J* = 8.5 Hz, 2H), 6.73 (d, *J* = 8.5 Hz, 2H), 6.70 (d, *J* = 8.0 Hz,1H), 6.68 (d, *J* = 2.0 Hz, 1H), 6.51 (dd, *J* = 8.0, 2.0 Hz,1H), 2.67 (m, 4H), 2.49 (m, 2H), 2.43 (m, 2H), 1.53 (m, 4H). ^13^C NMR (Acetone-*d*_6_, 125 MHz): *δ* 209.9, 156.4, 145.9, 144.1, 134.1, 134.0, 130.1, 120.4, 116.3, 116.0, 115.9, 45.0, 43.0, 35.5, 32.2, 29.9, 24.1. HR-ESI-MS *m/z*: [M + H]^+^ calcd for C_19_H_23_O_4_ 315.1591, found 315.1592.

### 4-(Methoxymethoxy)-1H-indole (10a)

To a solution of 4-hydroxyindole (100 mg, 0.75 mmol) in dry CH_2_Cl_2_ (10 ml) was added *N*,*N*-diisopropylethylamine (261 μl, 1.50 mmol) and chloromethyl methyl ether (MOMCl, 114 μl, 1.50 mmol) dropwise in an ice bath. The resultant was warmed to room temperature and stirred under N_2_ atmosphere for 2 h. The reaction mixture was diluted with distilled H_2_O (100 ml) and extracted with CH_2_Cl_2_ (3 × 50 ml). The combined organic layer was dried over anhydrous Na_2_SO_4_, filtered, and the solvent was concentrated *in vacuo*. The residue was purified over a silica gel column eluted with 11% EtOAc in *n*-hexane to give compound **10a** (89 mg, 67%) as a light yellow oil. ^1^H NMR (CDCl_3_, 300 MHz): *δ* 8.17 (br, 1H), 7.08 (m, 3H), 6.76 (dd, *J* = 7.1, 1.4 Hz, 1H), 6.66 (t, *J* = 2.7 Hz, 1H), 5.34 (s, 2H), 3.54 (s, 3H). ESI-MS *m/z*: 178.1 [M + H]^+^.

### 6-Benzyloxy-1H-indole (10b)

To a solution of 6-hydroxyindole (2.50 g, 18.78 mmol) and K_2_CO_3_ (12.98 g, 93.88 mmol) in dimethylformamide (DMF) (100 ml) was stirred under N_2_ atmosphere for 1 h. The solution was added benzyl chloride (4.32 ml, 37.55 mmol) dropwise in an ice bath. The resultant was warmed to room temperature and stirred under N_2_ atmosphere for 13 h. The reaction mixture was diluted with distilled H_2_O (100 ml) and extracted with EtOAc (5 × 100 ml). The combined organic layer was dried over anhydrous Na_2_SO_4_, filtered, and the solvent was concentrated *in vacuo*. The residue was purified over a silica gel column eluted with 10% EtOAc in *n*-hexane to give compound **10b** (3.31 g, 79%) as a yellow solid. Melting point 115–117 °C. ^1^H NMR (CDCl_3_, 300 MHz): *δ* 7.99 (br, 1H), 7.50 (d, *J* = 8.7 Hz, 1H), 7.45 (d, *J* = 6.9 Hz, 2H), 7.34 (m, 3H), 7.08 (dd, *J* = 3.2, 2.4 Hz, 1H), 6.93 (d, *J* = 2.2 Hz), 6.87 (dd, *J* = 8.7, 2.2 Hz, 1H), 6.47 (m, 1H), 5.10 (s, 2H). ESI-MS *m/z*: 224.11 [M + H]^+^.

### 6-(4-Methoxymethoxy-1H-indol-1-yl)hexan-2-one (11a)

To a solution of compound **10a** (100 mg, 0.56 mmol) in DMF (5 ml) was added NaH (82 mg, 3.40 mmol). The resulting solution was stirred for 1.5 h. The reaction mixture was added 6-chloro-hexanone (332 μl, 2.51 mmol) dropwise and was stirred at room temperature under N_2_ atmosphere for 24 h. The reaction mixture was diluted with distilled H_2_O (100 ml) and extracted with EtOAc (3 × 50 ml). The combined organic layer was dried over anhydrous Na_2_SO_4_, filtered, and the solvent was concentrated *in vacuo*. The residue was purified over a silica gel column eluted with 11% EtOAc in *n*-hexane to give compound **11a** (88 mg, 57%) as a brown oil. ^1^H NMR (CDCl_3_, 300 MHz): *δ* 7.09 (t, *J* = 8.0 Hz, 1H), 6.98 (m, 2H), 6.73 (d, *J* = 7.9 Hz, 1H), 6.57 (dd, *J* = 3.2, 0.7 Hz, 1H), 5.31 (s, 2H), 4.08 (t, *J* = 6.9 Hz, 2H), 3.52 (s, 3H), 2.38 (t, *J* = 7.2 Hz, 2H), 2.06 (s, 3H), 1.80 (m, 2H), 1.57 (m, 2H). ESI-MS *m/z*: 276.2 [M + H]^+^.

### 6-(6-Benzyloxy-1H-indol-1-yl)hexan-2-one (11b)

Following the procedure as described for the preparation of compound **11a**, the reaction of compound **10b** (100 mg, 0.45 mmol), NaH (43 mg, 1.79 mmol), 6-chloro-2-hexanone (176 μl, 1.33 mmol) in DMF (5 ml) gave compound **11b** (55 mg, 38%) as a brown solid. Melting point 43–46 °C. ^1^H NMR (CDCl_3_, 300 MHz): *δ* 7.47 (m, 3H), 7.33 (m, 3H), 6.96 (d, *J* = 3.2 Hz, 1H), 6.84 (m, 2H), 6.39 (dd, *J* = 3.2, 0.5 Hz, 1H), 5.11 (s, 2H), 4.02 (t, *J* = 7.1 Hz, 2H), 2.37 (t, *J* = 7.1 Hz, 2H), 2.37 (s, 3H), 1.77 (m, 2H), 1.58 (m, 2H). ESI-MS *m/z*: 322.2 [M + H]^+^.

### 6-(4-Hydroxy-1H-indol-1-yl)hexan-2-one (12a)

To a solution of compound **11a** (500 mg, 1.82 mmol) in MeOH (50 ml) at 0 °C was added 1 N HCl_(aq)_ (50 ml) dropwise. The resultant was warmed to room temperature and stirred under N_2_ for 22 h. The reaction mixture was diluted with distilled H_2_O (100 ml) and extracted with EtOAc (3 × 50 ml). The combined organic layer was dried over anhydrous Na_2_SO_4_, filtered, and the solvent was concentrated *in vacuo*. The residue was purified over a silica gel column eluted with 25% EtOAc in *n*-hexane to give compound **12a** (121 mg, 29%) as a yellow solid. Melting point 91–94 °C. ^1^H NMR (CDCl_3_, 300 MHz): *δ* 7.04 (t, *J* = 8.0 Hz, 1H), 6.99 (d, *J* = 3.2 Hz, 1H), 6.91 (d, *J* = 8.3 Hz, 1H), 6.49 (m, 2H), 5.11 (s, 1H), 4.08 (t, *J* = 6.9 Hz, 2H), 2.39 (t, *J* = 7.2 Hz, 2H), 2.07 (s, 3H), 1.81 (m, 2H), 1.57 (m, 2H). ESI-MS *m/z*: 232.1 [M + H]^+^.

### 6-(6-Hydroxy-1H-indol-1-yl)hexan-2-one (12b)

To a solution of compound **11b** (1.27 g, 3.95 mmol) and ammonium formate (2.49 g, 39.51 mmol) in MeOH (30 ml) was added catalysed 10% Pd–C (130 mg). The resultant was refluxed and stirred under N_2_ for 1.5 h. The reaction mixture was filtered through a celite pad. The residue was diluted with distilled H_2_O (100 ml) and extracted with EtOAc (3 × 50 ml). The combined organic layer was dried over anhydrous Na_2_SO_4_, filtered, and the solvent was concentrated *in vacuo*. The residue was purified over a silica gel column eluted with 28% EtOAc in *n*-hexane to give compound **12b** (329 mg, 36%) as a white solid. Melting point 94–98 °C. ^1^H NMR (CDCl_3_, 300 MHz): *δ* 7.42 (d, *J* = 8.1 Hz, 1H), 6.93 (d, *J* = 3.1 Hz, 1H), 6.75 (d, *J* = 2.2 Hz, 1H), 6.65 (dd, *J* = 8.1, 2.2 Hz, 1H), 6.38 (dd, *J* = 3.1, 0.8 Hz, 1H), 6.65 (s, 1H), 3.99 (t, *J* = 6.9 Hz, 1H), 2.39 (t, *J* = 7.1 Hz, 1H), 2.07 (s, 3H), 1.77 (m, 2H), 1.57 (m, 2H). ESI-MS *m/z*: 232.1 [M + H]^+^.

### (E)-1-(3,4-dihydroxyphenyl)-7-(4-hydroxy-1H-indol-1-yl)hept-1-en-3-one (13a)

Following the procedure as described for the preparation of compound **7a**, the reaction of compound **12a** (100 mg, 0.43 mmol) in THF (20 ml) with pyrrolidine (71 μl, 0.86 mmol), acetic acid (49 μl, 0.86 mmol), and 3,4- dihydroxybenzaldehyde (40 mg, 0.29 mmol) gave compound **13a** (40 mg, 39%) as a yellow solid. Melting point 180–184 °C. ^1^H NMR (Acetone-*d*_6_, 300 MHz): *δ* 8.34 (br, 3H), 7.46 (d, *J* = 16.2 Hz, 1H), 7.16 (m, 2H), 7.04 (dd, *J* = 8.2, 2.1 Hz, 1H), 6.95 (m, 2H), 6.87 (d, *J* = 8.2 Hz, 1H), 6.58 (m, 2H), 6.45 (dd, *J* = 2.8, 5.4 Hz, 1H), 4.18 (t, *J* = 7.2 H, 2H), 2.67 (dd, *J* = 7.2 Hz, 2H), 1.87 (m, 2H), 1.64 (m, 2H). ^13^C NMR (Acetone-*d*_6_, 125 MHz): *δ* 199.6, 151.6, 148.7, 146.3, 143.1, 139.0, 127.9, 127.0, 124.4, 122.9, 122.7, 119.5, 116.3, 115.2, 104.1, 102.4, 98.6, 46.7, 40.2, 30.5, 22.3. HR-ESI-MS *m/z*: [M + H]^+^ calcd for C_21_H_22_O_4_N 352.1543, found 352.1535.

### (E)-1-(3.4-dihydroxyphenyl)-7-(6-hydroxy-1H-indol-1-yl)hept-1-en-3-one (13b)

Following the procedure as described for the preparation of compound **7a**, the reaction of compound **12b** (50 mg, 0.22 mmol) in THF (5 ml) with pyrrolidine (24 μl, 0.29 mmol), acetic acid (17 μl, 0.29 mmol), and 3,4- dihydroxybenzaldehyde (19 mg, 0.14 mmol) gave compound **13b** (27 mg, 55%) as a brown solid. Melting point 155–159 °C. ^1^H NMR (Acetone-*d*_6_, 300 MHz): *δ* 8.32 (br, 2H), 7.92 (br, 1H), 7.46 (d, *J* = 16.2 Hz, 1H), 7.34 (d, *J* = 8.5 Hz, 1H), 7.17 (d, *J* = 2.1 Hz, 1H), 7.08 (d, *J* = 3.2 Hz, 1H), 7.04 (dd, *J* = 8.2 Hz, 2.0 Hz, 1H), 6.86 (m, 2H), 6.65 (dd, *J* = 8.5, 2.1 Hz, 1H), 6.58 (dd, *J* = 16.2 Hz, 1H), 6.31 (dd, *J* = 3.1, 0.6 Hz, 1H), 4.10 (t, *J* = 7.0 Hz, 2H), 2.67 (t. *J* = 7.3 Hz, 2H), 1.84 (m, 2H), 1.64 (m, 2H). ^13^C NMR (Acetone-*d*_6_, 125 MHz): *δ* 199.6, 154.1, 148.7, 146.3, 143.2, 138.0, 127.9, 127.3, 124.4, 123.3, 122.7, 121.8, 116.4, 115.2, 110.2, 101.4, 95.8, 46.4, 40.3, 30.4, 22.4. HR-ESI-MS *m/z*: [M + H]^+^ calcd for C_21_H_22_O_4_N 352.1543, found 352.1537.

### 1-(3,4-Dihydroxyphenyl)-7-(4-hydroxy-1H-indol-1-yl)heptan-3-one (14a)

Following the procedure as described for the preparation of compound **6a**, the reaction of compound **13a** (50 mg, 0.14 mmol) in EtOAc (10 ml) with 10% Pd–C (5 mg) gave compound **14a** (37 mg, 74%) as a black solid. ^1^H NMR (Acetone-*d*_6_, 300 MHz): *δ* 8.30 (s, 1H), 7.69 (s, 2H), 7.12 (d, *J* = 3.2 Hz, 1H), 6.94 (m, 2H), 6.70 (d, *J* = 8.0 Hz, 1H), 6.67 (d, *J* = 2.1 Hz,1H), 6.55 (d, *J* = 3.2 Hz, 1H), 6.50 (dd, *J* = 8.0, 2.1 Hz, 1H), 6.45 (dd, *J* = 7.0, 1.0 Hz, 1H), 4.12 (t, *J* = 7.0 Hz, 2H), 2.64 (m, 4H), 2.43 (t, *J* = 7.2 Hz, 2H), 1.77 (m, 2H), 1.52 (m, 2H). ^13^C NMR (Acetone-*d*_6_, 125 MHz): *δ* 209.6, 151.6, 145.7, 144.0, 139.0, 134.0, 127.0, 123.0, 120.2, 119.5, 116.2, 115.9, 104.1, 102.4, 98.6, 46.7, 44.9, 42.4, 30.4, 29.8, 21.6. HR-ESI-MS *m/z*: [M + H]^+^ calcd for C_21_H_24_O_4_N 354.1700, found 354.1696.

### 1-(3,4-Dihydroxyphenyl)-7-(6-hydroxy-1H-indol-1-yl)heptan-3-one (14b)

Following the procedure as described for the preparation of compound **6a**, the reaction of compound **13b** (68 mg, 0.19 mmol) in MeOH (5 ml) with 10% Pd–C (7 mg) gave compound **14b** (40 mg, 58%) as a brown oil. ^1^H NMR (Acetone-*d*_6_, 300 MHz): *δ* 7.89 (s, 1H), 7.66 (s, 1H), 7.63 (s, 1H), 7.33 (d, *J* = 8.5 Hz, 1H), 7.05 (d, 3.2 Hz, 1H), 6.81 (d, *J* = 2.1 Hz, 1H), 6.70 (d, *J* = 8.0 Hz, 1H), 6.67 (d, *J* = 2.1 Hz, 1H), 6.63 (dd, *J* = 8.5, 2.1 Hz, 1H), 6.50 (dd, *J* = 8.0, 2.1 Hz, 1H), 6.29 (dd, *J* = 3.2, 0.7 Hz, 1H), 4.06 (t, *J* = 7.0 Hz, 2H), 2.65 (m, 4H), 2.43 (t, *J* = 7.2 Hz, 2H), 1.76 (m, 2H), 1.53 (m, 2H). ^13^C NMR (Acetone-*d*_6_, 125 MHz): *δ* 209.6, 154.1, 145.7, 144.0, 138.0, 134.0, 127.3, 123.3, 121.8, 120.2, 116.2, 116.0, 110.2, 101.4, 95.8, 46.4, 44.9, 42.5, 21.6. HR-ESI-MS *m/z*: [M + H]^+^ calcd for C_21_H_24_O_4_N 354.1700, found 354.1695.

### (E)-1-(3,4-dihydroxyphenyl)-7-(4-(methoxymethoxy)-1H-indol-1-yl)hept-1-en-3-one (15)

Following the procedure as described for the preparation of compound **7a**, the reaction of compound **11a** (880 mg, 3.20 mmol) in THF (90 ml) with pyrrolidine (350 μl, 4.26 mmol), acetic acid (243 μl, 4.26 mmol), and 3,4-dihydroxybenzaldehyde (294 mg, 2.13 mmol) gave compound **15** (440 mg, 52%) as a yellow solid. Melting point 129–132 °C. ^1^H NMR (Acetone-*d*_6_, 300 MHz): *δ* 8.29 (br, 2H), 7.45 (d, *J* = 16.2 Hz,1H), 7.22 (d, *J* = 3.2 Hz, 1H), 7.15 (d, *J* = 2.1 Hz, 1H), 7.12 (m, 1H), 7.04 (m, 2H), 6.86 (d, *J* = 8.2 Hz, 1H), 6.69 (dd, *J* = 7.6, 0.8 Hz, 1H), 6.58 (d, *J* = 16.2 Hz, 1H), 6.51 (dd, *J* = 3.2, 0.7 Hz, 1H), 5.30 (s, 2H), 4.22 (t, *J* = 7.0 Hz, 2H), 3.47 (s, 3H), 2.68 (t, *J* = 7.3 Hz, 2H), 1.88 (m, 2H), 1.64 (m, 2H). ^13^C NMR (Acetone-*d*_6_, 125 MHz): *δ* 199.6, 151.7, 148.7, 146.3, 143.1, 138.7, 127.9, 127.7, 124.4, 122.7, 122.6, 120.9, 116.4, 115.2, 104.8, 104.0, 98.7, 95.4, 56.0, 46.7, 40.2, 30.6, 22.3. HR-ESI-MS *m/z* [M + H]^+^ calcd for C_23_H_26_O_5_N 396.1805, found 396.1978.

### 6-(1H-indol-1-yl)hexan-2-one (17)

Following the procedure as described for the preparation of compound **11a**, the reaction of indole (100 mg, 0.85 mmol) and NaH (82 mg, 3.42 mmol) in DMF (5 ml) with 6-chloro-2-hexanone (332 μl, 2.55 mmol) gave compound **17** (93 mg, 51%) as a yellow oil. ^1^H NMR (CDCl_3_, 300 MHz): *δ* 7.61 (m, 1H), 7.32 (dd, *J* = 8.2, 0.8 Hz, 1H), 7.19 (m, 1H), 7.08 (m, 2H), 6.47 (dd, *J* = 3.1, 0.8 Hz, 1H), 4.11 (t, *J* = 6.9 Hz, 2H), 2.39 (t, *J* = 7.2 Hz, 2H), 1.82 (m, 2H), 1.57 (m, 2H). ESI-MS *m/z*: 216.1 [M + H]^+^.

### (E)-1-(3,4-dihydroxyphenyl)-7-(1H-indol-1-yl)hept-1-en-3-one (18)

Following the procedure as described for the preparation of compound **7a**, the reaction of compound **17** (100 mg, 0.46 mmol) in THF (10 ml), pyrrolidine (50 μl, 0.61 mmol), acetic acid (35 μl, 0.61 mmol), and 3,4-dihydroxybenzaldehyde (43 mg, 0.31 mmol) gave compound **18** (81 mg, 78%) as a orange solid. Melting point 165–169 °C. ^1^H NMR (DMSO-*d*_6_, 300 MHz): *δ* 9.28 (br, 2H), 7.53 (d, *J* = 7.8 Hz, 1H), 7.47 (d, *J* = 8.1 Hz, 1H), 7.41 (d, *J* = 16.2 Hz, 1H), 7.37 (d, *J* = 3.2 Hz, 1H), 7.11 (m, 1H), 7.05 (d, *J* = 2.0 Hz, 1H), 6.99 (m, 2H), 6.76 (d, *J* = 8.1 Hz, 1H), 6.51 (d, *J* = 16.2 Hz, 1H), 6.41 (dd, *J* = 3.2, 0.6 Hz, 1H), 4.19 (t, *J* = 6.9 Hz, 2H), 2.65 (t, *J* = 7.1 Hz, 2H), 1.77 (m, 2H), 1.50 (m, 2H). ^13^C NMR (Acetone-*d*_6_, 125 MHz): *δ* 199.5, 148.7, 146.3, 143.1, 137.0, 129.7, 128.9, 127.9, 124.4, 122.7, 121.8, 121.4, 119.7, 116.4, 115.2, 110.4, 101.4, 46.5, 40.2, 30.6, 22.3. HR-ESI-MS *m/z*: [M + H]^+^ calcd for C_21_H_22_O_3_N 336.1594, found 336.1587.

### 1-(3,4-Dihydroxyphenyl)-7-(1H-indol-1-yl)heptan-3-one (19)

Following the procedure as described for the preparation of compound **6a**, the reaction of compound **18** (226 mg, 0.67 mmol) in EtOAc (25 ml) with 10% Pd–C (23 mg) gave compound **19** (120 mg, 53%) as a white solid. Melting point 110–113 °C. ^1^H NMR (Acetone-*d*_6_, 300 MHz): *δ* 7.66 (s, 1H), 7.63 (s, 1H), 7.54 (m, 1H), 7.42 (dd, *J* = 8.2, 0.8 Hz, 1H), 7.26 (d, *J* = 3.2 Hz, 1H), 7.13 (ddd, *J =* 8.2, 7.2, 1.1 Hz, 1H), 7.00 (ddd, *J* = 7.9, 7.0, 0.8 Hz, 1H), 6.70 (d, *J* = 8.1 Hz, 1H), 6.66 (d, *J* = 2.1 Hz, 1H), 6.50 (dd, *J* = 7.9, 2.1 Hz, 1H), 6.42 (dd, *J* = 3.2, 0.8 Hz, 1H), 4.19 (t, *J* = 7.0 Hz, 2H), 2.65 (m, 4H), 2.44 (t, *J* = 7.2 Hz, 2H), 1.80 (m, 2H), 1.53 (m, 2H). ^13^C NMR (Acetone-*d*_6_, 125 MHz): *δ* 209.5, 145.7, 144.0, 137.0, 134.0, 129.6, 128.9, 121.9, 121.4, 120.2, 119.7, 116.2, 115.9, 110.3, 46.5, 44.7, 42.4, 30.4, 29.8, 21.6. HR-ESI-MS *m/z*: [M + H]^+^ calcd. for C_21_H_24_O_3_N, 338.1751, found 338.1744.

### Molecular docking analysis

The docking software LeadIT^40^ (version 2.3.2) was utilised to analyse the interactions between the compounds and LIMK1 (PDB ID: 3S95). The co-crystal structure of LIMK1 was obtained from the Protein Data Bank (PDB). The location of the co-crystallised ligand was used to define the binding site, which was defined with a radius of 10 Å. Water molecules were removed from the binding site prior to analysis. All docked compounds were protonated in an aqueous solution. The FlexX docking module of LeadIT was employed to conduct the docking analysis. Ligand placement is guided by a hybrid enthalpy–entropy approach, ensuring that both energetic and entropic contributions are considered when evaluating binding interactions. In the scoring section, access scaling is enabled, applying uniform settings to all hydrogen bond–relevant contact types. The thresholds are set so that contacts contribute fully at 0.30 and contribute no score at 0.70 or higher. For clash handling, the system allows a maximum protein–ligand overlap volume of 2.9 Å³, indicating a moderate tolerance, while intra-ligand clashes are controlled with a clash factor of 0.6. Hydrogens are included in internal clash evaluations for greater accuracy. Finally, the docking details specify that up to 200 solutions per iteration and 200 solutions per fragmentation will be generated, which provides a relatively broad sampling of conformational space. Overall, this configuration balances flexibility with efficiency, applying moderate clash stringency while enabling extensive exploration of possible ligand poses.

### Kinase inhibition assay

The inhibitory activities of the compounds against kinases were tested using the SelectScreen Kinase assay from Thermo Fisher Scientific in Waltham, MA, USA. The LanthaScreen Eu kinase binding assay was used to validate the LIMK1 inhibition activity of the compounds, and was performed according to the manufacturer’s protocol (https://www.thermofisher.com/tw/zt/home/industrial/pharma-biopharma/drug-discovery-development/target-and-lead-identification-and-validation/kinasebiology/kinase-activity-assays/lanthascreentm-eu-kinase-binding-assay/lanthascreen-eu-kinase-binding-assay-validation-table.html). This endpoint assay quantifies inhibitor binding through the displacement of a fluorescently labelled ATP-competitive tracer from LIMK1. A mixture of the 100× compounds, 2× LIMK1/antibody mixture (Eu-anti-His), 4× tracer 236, and kinase buffer were mixed sequentially. After incubation at room temperature for 1 h, the mixture was analysed using a fluorescence plate reader. The kinase inhibition assay results were expressed as the average of two replicates.

Kinase panel screening was carried out using the SelectScreen Kinase Assay (Thermo Fisher Scientific, Waltham, MA, USA). Three complementary assay formats were applied: the Z′-LYTE kinase assay, LanthaScreen Eu kinase binding assay, and Adapta Universal kinase assay. The kinases selected for this study and the corresponding assay formats are summarised in Supplementary Table S1.

The Z′-LYTE Kinase Assay (Thermo Fisher Scientific, Waltham, MA, USA) was used to evaluate selected kinases’ activity, according to the manufacturer’s instructions (https://www.thermofisher.com/tw/zt/home/industrial/pharma-biopharma/drug-discovery-development/target-and-lead-identification-and-validation/kinasebiology/kinase-activity-assays/z-lyte/z-lyte-reactivity-table.html). This endpoint assay quantifies kinase activity by detecting peptide substrate phosphorylation. Briefly, a 2× mixture of selected kinase and relevant substrate was prepared in kinase buffer (0.01% polyoxyethylene lauryl ether (BRIJ-35), 50 mM 4-(2-hydroxyethyl)-1-piperazineethanesulfonic acid (HEPES), pH 7.5, 1 mM ethylene glycol-bis(β-aminoethyl ether)-N,N,N′,N′-tetraacetic acid (EGTA), and 10 mM MgCl_2_). After 1 h incubation at room temperature, 5 μl of 1:128 diluted development reagent solution was added, followed by an additional 1 h incubation. Fluorescence was recorded using a multimode plate reader.

The Adapta Universal Kinase Assay (Thermo Fisher Scientific, Waltham, MA, USA) was used to assess kinase activity. This homogeneous, fluorescence-based immunoassay quantifies kinase activity by detecting ADP produced during the enzymatic reaction, according to the manufacturer’s instructions (https://www.thermofisher.com/tw/zt/home/industrial/pharma-biopharma/drug-discovery-development/target-and-lead-identification-and-validation/kinasebiology/kinase-activity-assays/adapta-universal-kinase-assay.html).

### Cell culture

Colorectal cell line, HCT-116, was purchased from the Bioresource Collection and Research Centre (BCRC, Hsinchu, Taiwan), while HT-29 was obtained from the American Type Culture Collection (ATCC, Manassas, VA, USA). Both cell lines were maintained in McCoy’s 5 A medium (with L-glutamine and 2.2 g/L NaHCO_3_, M4892, Sigma) supplemented with 10% foetal bovine serum (Corning Incorporated, NY, USA) and 1% penicillin–streptomycin–amphotericin B (100 unit/ml penicillin G, 0.1 mg/ml streptomycin sulphate, 0.25 μg/ml amphotericin B, Corning Incorporated, NY, USA). All cultures were maintained in a humidified incubator at 37 °C with 5% CO_2_.

### MTT assay

Cells were seeded in 96-well plates at a density of 3 × 10^3^ cells/well in 100 μl of culture medium and treated with compounds at the indicated concentrations (0.3, 1, 3, 10, and 30 μM) for 72 h. Following treatment, the 3-(4,5-dimethylthiazol-2-yl)-2,5-diphenyltetrazolium bromide (MTT) solution (5 mg/ml in Phosphate-buffered saline (PBS)) was added to each well to reach a final concentration of 0.5 mg/ml, and the cells were incubated at 37 °C for 1 h. The resulting formazan crystal was dissolved in 100 µl of DMSO, and the absorbance was measured at 550 nm using an ELISA reader (Molecular Device, Sunnyvale, CA, USA).

### Sulforhodamine B assay

Cells were seeded in 96-well plates at a density of 3 × 10^3^ cells/well in 100 μl of culture medium and treated with compounds at the indicated concentrations for 72 h. After treatment, cells were fixed with 10% trichloroacetic acid for 20 min and washed with H_2_O. The cells were then stained with 0.4% sulforhodamine B (SRB) in 1% acetic acid for 20 min, followed by two washes with 1% acetic acid. After the well-dried, the protein-bound dye was dissolved in 100 µl of 10 mM Tris base solution, and the absorbance was measured at 515 nm using an ELISA reader (Molecular Device, Sunnyvale, CA, USA).

### Analysis of cell cycle by flow cytometry

Cells were seeded in six-well plates at a density of 5 × 10^5^ cells/well in a 2 ml culture medium and treated with compounds at the indicated concentration for 72 h. After treatment, cells were collected and fixed with 75% (v/v) ice-cold ethanol at −20 °C for 20 min. The cells were then centrifuged to remove the buffer, resuspended, and stained with 0.5 ml of propidium iodide (PI) staining buffer (80 µg/ml PI, 100 µl/ml RNase A, and 1% Triton X-100 in PBS). The cell cycle distribution was analysed using a BD Accuri™ Flow cytometer and software (Becton Dickinson, Mountain View, CA, USA).

### Statistical analysis

All biological experiments were repeated at least three times to ensure reproducibility and reliability. Data are presented as the mean ± standard deviation (SD) or expressed as a percentage of the control group, depending on the nature of the experiment. Statistical analyses were performed using a one-way analysis of variance (non-parametric test) in GraphPad Prism. A *p* values of <0.05 was considered statistically significant.

## Result and discussion

### Design rationale

**Figure 2.**
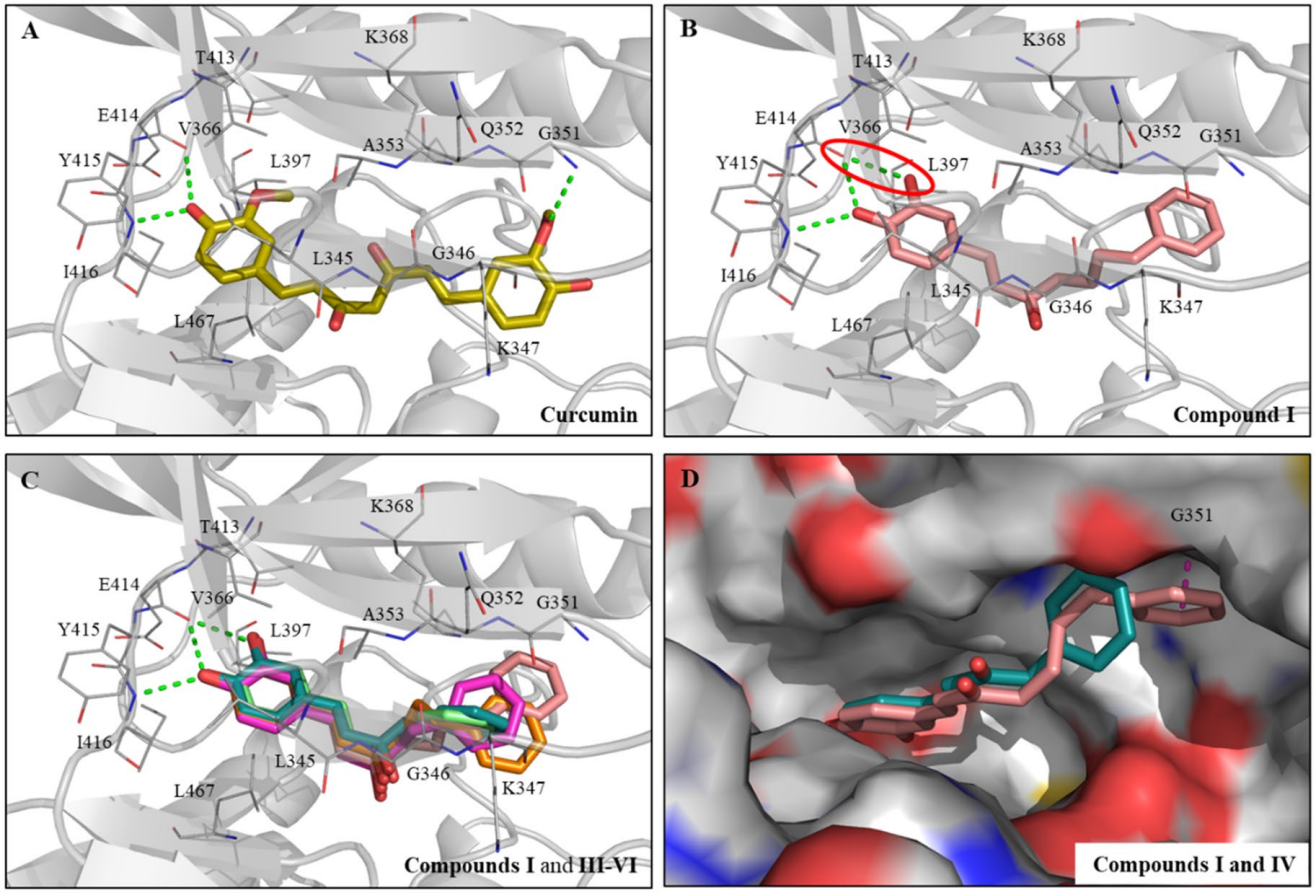
Interaction analysis of curcumin, compounds **I** and **III**–**VI** with LIMK1 binding site. Docking poses of (A) curcumin (yellow) and (B) compound **I** (salmon) in the LIMK1 binding site (PDB ID: 3S95). (C) Superimposed docking poses of compounds **I**, **III** (lime-green), **IV** (blue-green), **V** (magenta), and **VI** (orange). (D) Docking pose of compound **I** superimposed onto the pose of compound **IV** in LIMK1 (surface model). Hydrogen bonds are represented as green dashed lines.

A previous study found that curcumin has LIMK inhibitory activity^37^. To better understand the interactions between curcumin and the LIMK1 binding site, we conducted a molecular docking analysis. The docking results showed that curcumin occupies the binding site (Figure 2(A)). Specifically, the 4-hydroxy-3-methoxyphenyl group of curcumin acts as a hinge binder, forming two hydrogen bonds with hinge residues E414 and I416. In addition, the aromatic ring of curcumin interacts with the LIMK1 binding site through π-alkyl interactions with residues L345, V366, and L467. Two aromatic rings of curcumin are attached together through a seven-carbon length and extend into another pocket of the binding site. This group contributes an additional hydrogen bond with residue G351. These observed interactions suggest that the inhibitory activity of curcumin is due to its interaction with LIMK1, indicating diarylheptanoid as a promising scaffold for a LIMK1 inhibitor.

Compound **I** can be classified as a diarylheptanoid, which was designed and synthesised in our previous research^41^. The catechol group of compound **I** might act as a hydrogen bond donor or acceptor simultaneously, which is advantageous for interacting with hinge residues. Consequently, compound **I** was evaluated for its ability to inhibit LIMK1, and the result indicated stronger activity compared to curcumin, with an IC_50_ value of 1.59 µM (Table 1). The docking result revealed that compound **I** forms more hydrogen bonds with hinge residues than curcumin, primarily due to the presence of the catechol group (Figure 2(B)). Specifically, compound **I** forms an additional hydrogen bond with hinge residue V366 through the 3-hydroxy group (Figure 2(B)), which may explain its superior LIMK1 inhibition compared to curcumin. Therefore, the catechol has the potential to serve as a hinge binder in the design of a new series of diarylheptanoid-based LIMK1 inhibitors.

**Table 1.** Inhibitions of compounds **I**–**VI** against LIMK1.

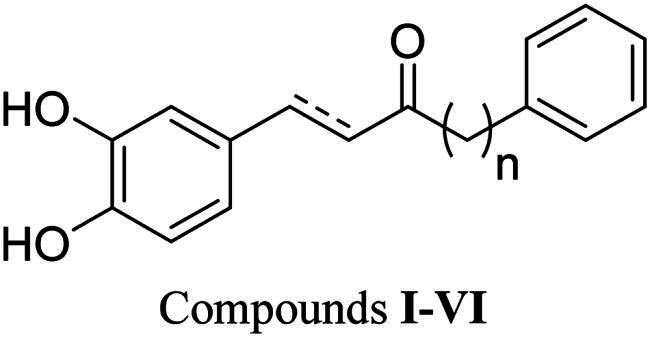			
Compound	Chain length (*n*)	α, β-saturation	Inhibition percentage at 10 μM (IC_50_)
**I**	4	Unsaturated	101% (1.59 µM)
**II**	4	Saturated	30%
**III**	0	Unsaturated	84%
**IV**	1	Unsaturated	56%
**V**	2	Unsaturated	68%
**VI**	3	Unsaturated	82%

To further investigate the impact of the α-β saturation on LIMK1 inhibitory activity, compound **II**, which has a saturated linker, was compared to compound **I**. The result showed that saturated compound **II** only exhibited 30% inhibition at 10 μM (Table 1), suggesting the importance of the unsaturated bond for LIMK1 inhibition. Subsequently, unsaturated compounds **III**–**VI** were evaluated for their LIMK1 inhibition at 10 μM to study the effects of carbon chain length. The experimental results revealed that compounds with shorter carbon chains did not demonstrate better LIMK1 inhibition than compound **I** (Table 1). Docking results of the compounds indicated similar interactions in the hinge region (Figure 2(C)). However, their B ring contributed to different interactions with LIMK1 residues due to varying linker lengths. Among these compounds, compound **IV** showed less potency in LIMK1 inhibition. The docking pose of compound **IV** in LIMK1 was compared to that of compound **I** (Figure 2(D)). It was found that the chain shortening of compound **III** resulted in its B ring not being able to extend into another pocket containing residue G351, possibly accounting for its weaker potency.

In short, we found that catechol-containing diarylheptanoid is a suitable scaffold with stronger inhibitory activity than curcumin. To enhance the inhibitory activity, we selected compound **I** as a lead compound and synthesised a series of catechol-containing diarylheptanoid derivatives. Specifically, we added a hydroxy group to the B ring to create compounds **7a**–**c** and **8a**–**c**, aiming to form additional hydrogen bonds. Moreover, to achieve extra π interaction with LIMK1, we developed compounds **13a, 13b**, **14a, 14b**, **15**, **18**, and **19** by replacing the B ring with indole. These changes in structure are expected to enhance the LIMK1 inhibitory activity.

## Chemistry

### Synthesis of compounds 7a–c and 8a–c

**Scheme 1.**
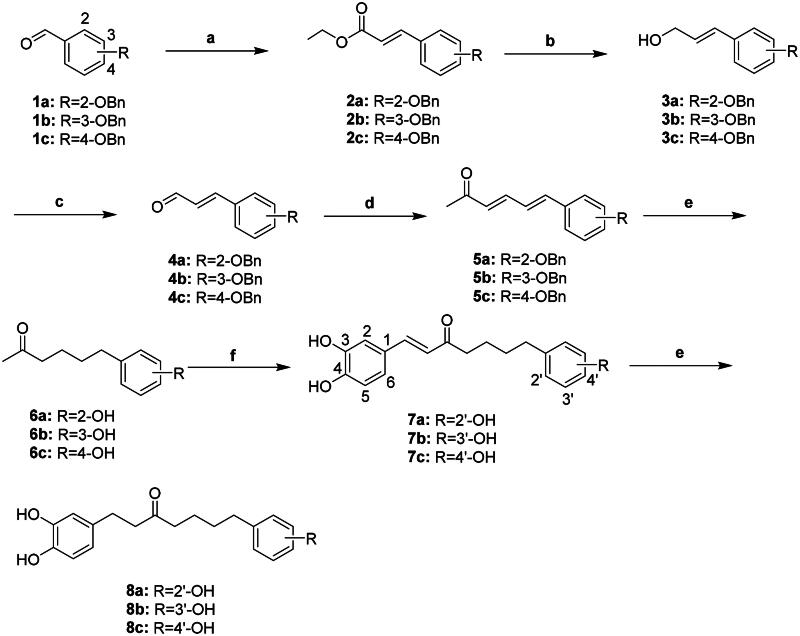
Reagents and conditions: (a) Triethyl phosphonoacetate, NaH, dry THF, 0 °C; (b) DIBAL-H, dry CH_2_Cl_2_, −30 °C; (c) MnO_2_, toluene, 110 °C; (d) 1-Triphenylphosphoranylidene-2-propanone, dry toluene, 110 °C; (e) 10% Pd–C, H_2_, EtOH, RT; and (f) 3, 4-dihydroxybenzaldehyde, pyrrolidine, AcOH, THF, 66 °C.

Compounds **7a–c** and **8a–c** were synthesised as shown in Scheme 1. Initially, the Horner–Wadsworth–Emmons reaction of benzyl-protected benzaldehydes **1a–c** with triethyl phosphonoacetate in the presence of NaH provided compounds **2a–c**, respectively. Reduction of compounds **2a–c** with DIBAL-H generated corresponding alcohols **3a–c**. Oxidation of compounds **3a–c** using MnO_2_ produced aldehydes **4a–c,** respectively. Wittig reaction of compounds **4a–c** with 1-(triphenylphosphoranylidene)-1-propanone yielded compounds **5a–c**. Atmospheric hydrogenation of compounds **5a–c** using catalytic 10% Pd–C removed the benzyl protecting group, producing the corresponding compounds **6a–c**. Aldol condensation of compounds **6a–c** with 3, 4-dihydroxybenzaldehyde generated compounds **7a–c**. Repeated hydrogenation of compounds **7a–c** gave saturated compounds **8a–c,** respectively.

### Synthesis of compounds 13a, 13b, 14a, 14b, 15, 18, and 19

**Scheme 2.**
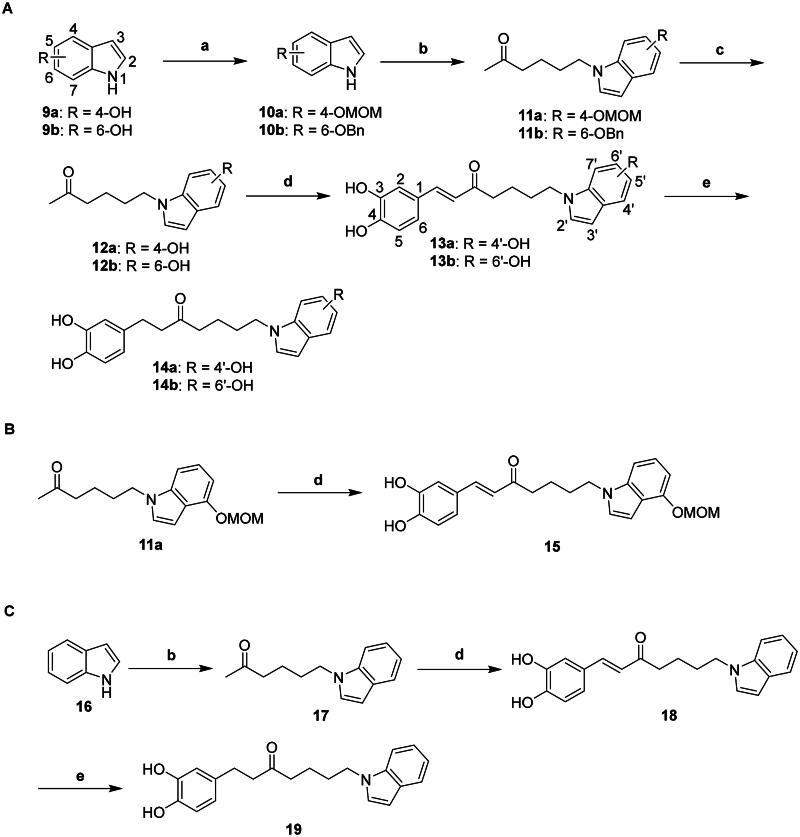
Reagents and conditions: (a) for **10a**: MOMCl, DIPEA, CH_2_Cl_2_, RT; for **10b**: BnCl, K_2_CO_3_, DMF, 80 °C**; (b) 6**-chloro-2-hexanone, NaH, DMF, RT; (c) for **12a**: 1 N HCl_(aq)_, MeOH, RT; for **12b**: 10% Pd–C, NH_4_HCO_2_, MeOH, 80 °C; (d) 3,4-dihydroxybenzaldehyde, pyrrolidine, acetic acid, THF, RT; and (e) 10% Pd–C, H_2_, EtOAc, RT.

Compounds **13a, 13b, 14a, 14b, 15, 18**, and **19** were synthesised as outlined in Scheme 2. The hydroxy groups of indoles **9a** and **9b** were protected by reacting with chloromethyl methyl ether (MOMCl) and benzyl chloride (BnCl) to produce compounds **10a** and **10b**, respectively. Subsequent nucleophilic substitution of compounds **10a** and **10b** with 6-chloro-2-hexanone generated corresponding compounds **11a** and **11b**. Deprotection of compounds **11a** and **11b** yielded compounds **12a** and **12b**, which then underwent aldol condensation with 3,4-dihydroxybenzaldehyde to produce compounds **13a** and **13b**. Atmospheric hydrogenation of compounds **13a** and **13b** yielded compounds **14a** and **14b,** respectively. Repeated aldol condensation of compound **11a** afforded compound **15**. The synthetic approach for compound **19** was similar to that for compounds **14a** and **14b**. All compounds tested for inhibitory activity were further confirmed for their molecular composition by high-resolution mass spectrometry. Detailed characterisation spectra (^1^H and ^13^C NMR) for the synthesised compounds are provided in both the “Materials and Methods” section and the Supplementary Materials.

## Biological evaluation

### *Inhibition of compounds 7a–c, 8a*–*c, 13a, 13b, 14a, 14b, 15, 18, and 19 against LIMK1*

**Figure 3.**
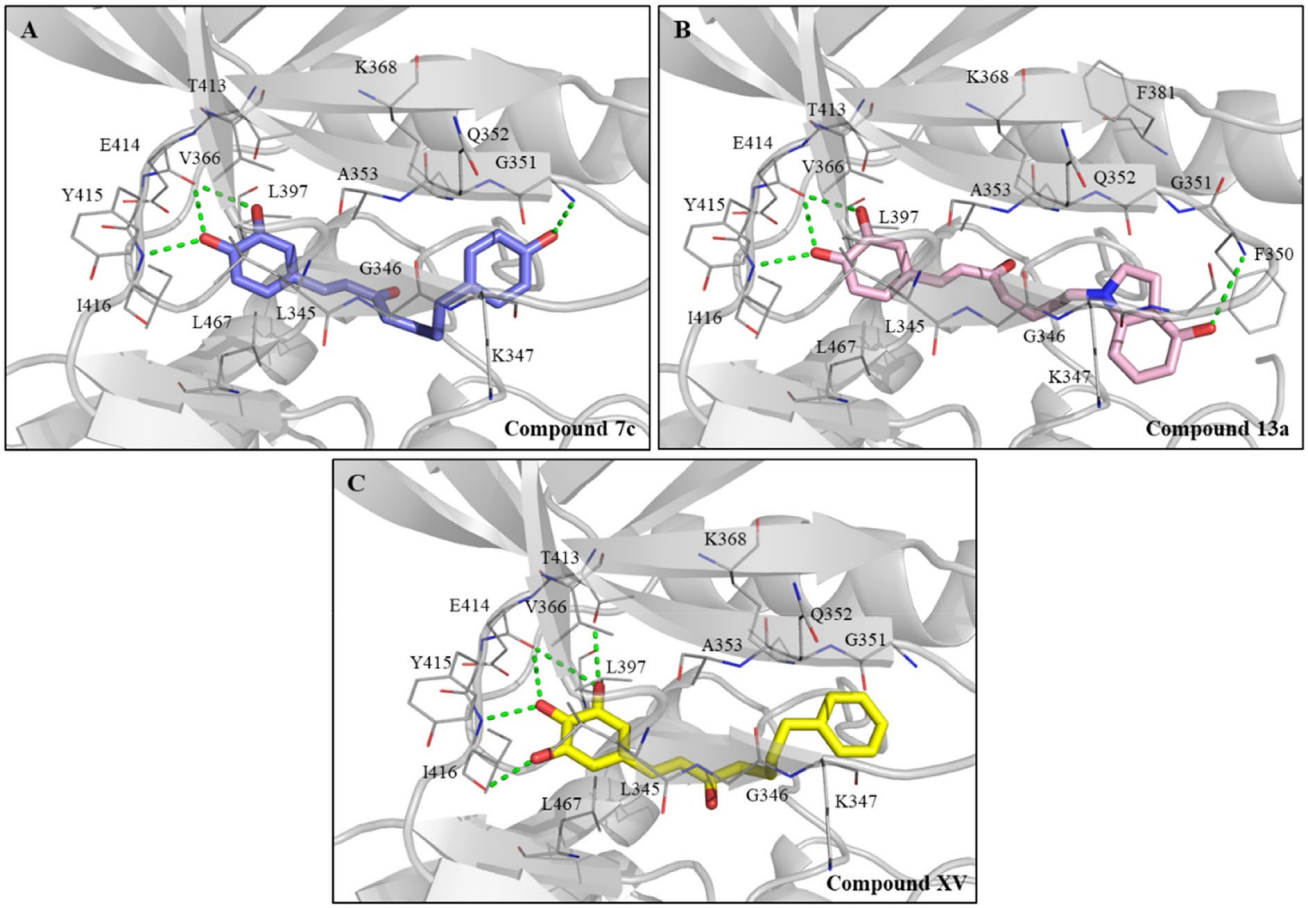
Molecular docking analysis of compounds **7c**, **13a**, and **XV** in LIMK1. Docking poses of (A) compound **7c** (slate), (B) compound **13a** (pink), and (C) compound **XV** (yellow) with LIMK1 (grey). The residues involved in the interaction are labelled as depicted. Hydrogen bonds are depicted as green dashed lines.

The synthesised compounds were evaluated for their ability to inhibit LIMK1. The percentage of LIMK1 inhibition for each compound was measured at a concentration of 10 μM (Table 2). Compounds that exhibited over 80% inhibitory activity at this concentration were chosen for further evaluation of their IC_50_ values. Similar to the previously mentioned results, α-β unsaturated compounds **7a–c**, **13a**, **13b**, and **18** showed greater potency compared to the saturated compounds **8a–c**, **14a, 14b**, and **19**. With compounds **7a–c**, compound **7c,** which had a 4′-hydroxy substitution, displayed higher LIMK1 inhibition compared to compounds with 3′ and 2′-hydroxy substitution. The docking result suggested that the improvement may be attributed to the formation of an additional hydrogen bond between residue G351 and the 4′-hydroxy group of compound **7c** (Figure 3(A)). In the compounds containing an indole group, compound **13a** with a 4′-hydroxy group showed better LIMK1 inhibitory activity than compound **13b** with a 6′-hydroxy group. The 4′-hydroxy group of compound **13a** forms a hydrogen bond with residue F350 (Figure 3(B)). Compounds **15** and **18**, which lacked a hydroxy group on the indole, exhibited lower potency than compound **13a**. Additionally, among these compounds tested, compound **13a** demonstrated the most potent LIMK1 inhibition. These results also suggested that the incorporation of an additional hydroxy group in the B ring/indole and the substitution of the B ring with indole enhance the LIMK1 inhibitory activity of the compounds.

**Table 2.** Inhibitions of compounds **7a–c**, **8a–c**, **13a, 13b**, **14a, 14b**, **15**, **18,** and **19** against LIMK1.

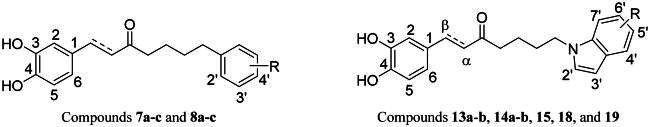				
Compound	Substitution (R)	α, β-saturation	Inhibition percentage at 10 μM	IC_50_
**7a**	2′-OH	Unsaturated	89%	1.77 µM
**7b**	3′-OH	Unsaturated	102%	1.11 µM
**7c**	4′-OH	Unsaturated	106%	1.09 µM
**8a**	2′-OH	Saturated	37%	N.D.^a^
**8b**	3′-OH	Saturated	34%	N.D.
**8c**	4′-OH	Saturated	47%	N.D.
**13a**	4′-OH	Unsaturated	109%	0.94 µM
**13b**	6′-OH	Unsaturated	106%	1.39 µM
**14a**	4′-OH	Saturated	51%	N.D.
**14b**	6′-OH	Saturated	53%	N.D.
**15**	4′-OMOM	Unsaturated	106%	1.88 µM
**18**	H	Unsaturated	110%	1.49 µM
**19**	H	Saturated	28%	N.D.

^a^ N.D.: not determined.

### Inhibition of compounds VII–XV against LIMK1

The impact of the hydroxy group on A ring against LIMK1 was investigated using nine previously synthesised compounds **VII–XV** (Table 3). Compound **VII**, which was without hydroxy groups, and compounds **VIII–IX**, which had only one hydroxy group, lost their inhibitory activity. Additionally, compounds with an electron-withdrawing group at the 3-position (compounds **X**–**XII**) or an electron-donating group (compound **XIII**) exhibited reduced inhibitory activity. However, compound **XV**, which consists of a pyrogallol group, showed the most potent LIMK1 inhibition with an IC_50_ value of 569 nM. Similar to the catechol group of compound **I**, compound **XV** also formed the same three hydrogen bonds with hinge residues E414 and I416 (Figure 3(B)). Additionally, the 3,4,5-trihydroxy group of compound **XV** established an extra hydrogen bond with residue I416. Furthermore, compound **XV** formed another hydrogen bond with residue T413 near the hinge region. In total, five hydrogen bonds were formed between compound **XV** and LIMK1. The polar interaction allows compound **XV** to bind with LIMK1 and stabilise it. These findings suggest that having at least two hydroxy groups on the A ring is essential for LIMK1 inhibition. The above results provide valuable information for constructing SAR.

**Table 3.** Inhibitions of compounds **VII–XV** against LIMK1.

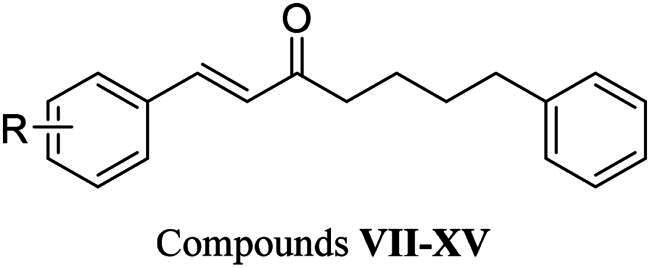		
Compound	Substitution (R)	Inhibition percentage at 10 μM (_IC50_)
**VII**	H	≤0%
**VIII**	4-OH	≤0%
**IX**	3-OH	≤0%
**X**	3-F, 4-OH	≤0%
**XI**	3-Cl, 4-OH	4%
**XII**	3-Br, 4-OH	1%
**XIII**	3-NO_2_, 4-OH	7%
**XIV**	3-OCH_3_, 4-OH	1%
**XV**	3,4,5-OH	112% (0.57 µM)

To elucidate the effect of saturated and unsaturated compounds, compound **S** was designed to compare with compound **I**. As shown in Supplementary Figure S48(A), both compounds share a common caffeoyl moiety (Group A), whereas Group B differs by the presence of an unsaturated chain in compound **S** and a saturated chain in compound **I**. The 2D interaction mapping (Supplementary Figure S48(B)) revealed that Group A consistently forms hydrogen bonds with residues E414 and I416. These interactions suggest that the caffeoyl group functions as a conserved anchoring pharmacophore responsible for stable binding within the LIMK1 binding site.

Superimposed docking poses (Supplementary Figure S48(C)) further demonstrated that both compounds adopt similar orientations in Group A, whereas the structural divergence of Group B leads to distinct conformational adaptations. The unsaturated chain of compound **S** is positioned in closer proximity to the hydrophobic cavity near G351, which may promote additional interactions. In contrast, the saturated chain of compound **I** provides greater conformational flexibility. Taken together, these findings indicate that while Group A ensures a conserved interaction motif across both compounds, Group B critically modulates binding affinity.

### Kinase selectivity of compound 13a

**Figure 4.**
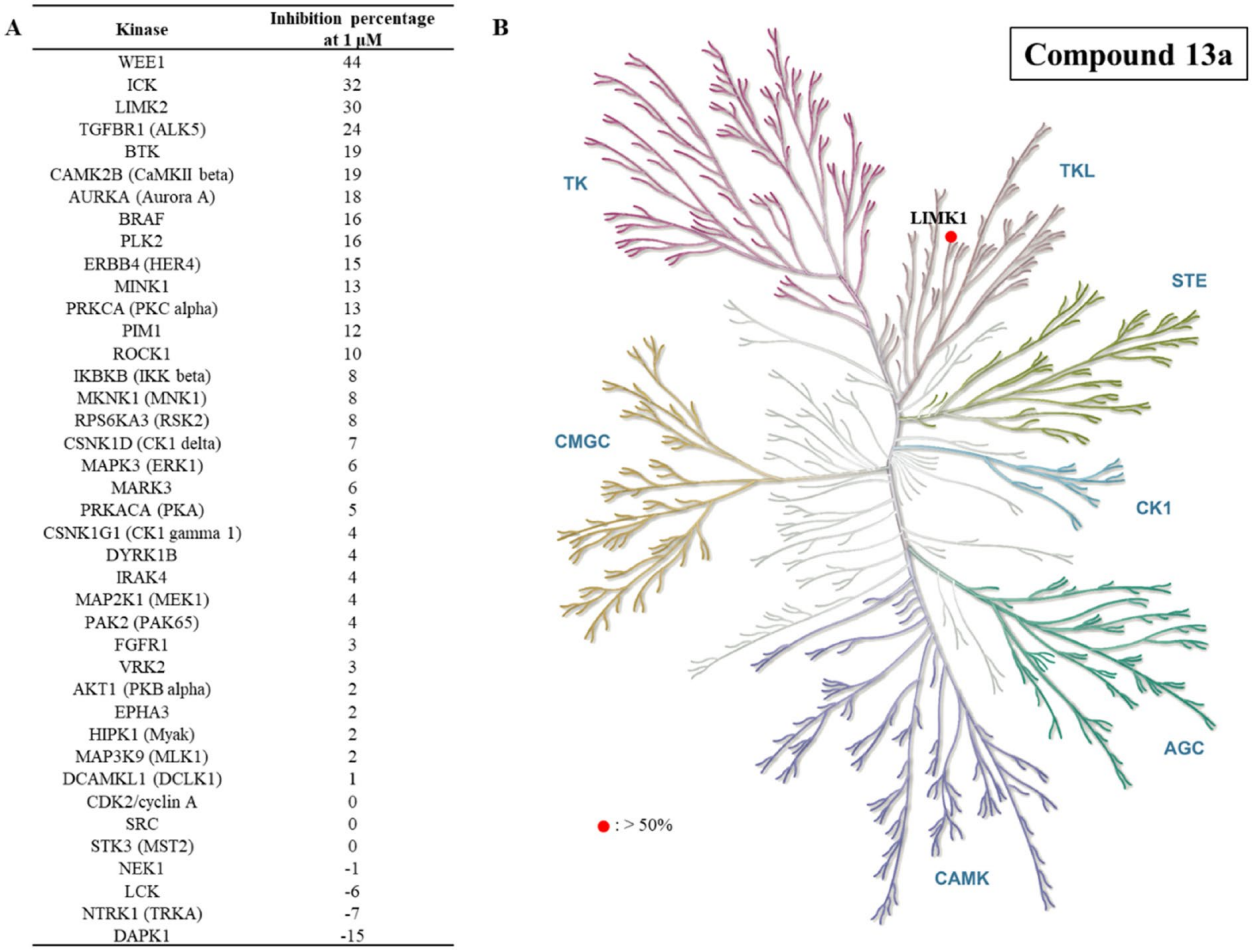
Inhibition activity of compound **13a** against a panel of representative kinases.

To evaluate the kinase selectivity of compound **13a**, a panel of 40 kinases from various families was selected to determine its inhibitory selectivity further (Figure 4). The inhibition percentages for compound **13a** across these kinases were all below 50% at a concentration of 1 μM. Specifically, it exhibited 44%, 32%, and 30% inhibition percentages for WEE1, ICK, and LIMK2, respectively. Notably, compound **13a** demonstrated lower potency in inhibiting LIMK2 compared to LIMK1. A decrease in LIMK2 levels results in an increased accumulation of β-catenin in the nucleus, which activates the Wnt signalling pathway^9^. This activation is crucial for advancing CRC progression^9^. Therefore, the selective inhibition of LIMK1 over LIMK2 emphasises the advantages of compound **13a**. Besides that, LIMK1 belongs to the tyrosine kinase-like (TKL) kinase family, and compound **13a** displayed selectivity for other TKL family, including IRAK4, MLK1, BRAF, and TGFBR1. Given the high homology among the catalytic domains of kinases, most ATP-binding inhibitors can unintentionally affect multiple kinases, potentially leading to unwanted side effects. Fortunately, the selectivity analysis results indicate that compound **13a** shows a preference for inhibiting LIMK1 (Figure 4).

The pharmacokinetic and drug-likeness properties of compound **13a** were evaluated using the ADMETlab 3.0^42^ platform. As summarised in Supplementary Table S2, compound **13a** displays physicochemical, absorption, distribution, metabolism, and excretion (ADME) profiles characteristic of drug-like small molecules. Compound **13a** possesses a molecular weight of 351.15, a logP value of 2.95, and a topological polar surface area (TPSA) of 82.69 Å^2^, all within the optimal range for small-molecule drugs, suggesting a favourable balance between membrane permeability and aqueous solubility. Furthermore, compound **13a** complies with multiple medicinal chemistry filters, including Lipinski’s Rule of Five, Pfizer, GSK, and Golden Triangle criteria, collectively supporting its overall drug-likeness.

Regarding ADME (Supplementary Table S2), the predicted permeability profiles revealed that the Caco-2 permeability (−5.091) was within the acceptable range for permeable compounds, and the compound is predicted to be a non-substrate of P-glycoprotein (0.02), suggesting that its cellular uptake is unlikely to be restricted by efflux transporters. Predicted human intestinal absorption (0.038) also indicates possible oral absorption. Additionally, compound **13a** demonstrates high plasma protein binding (95.385%), low blood–brain barrier (BBB) penetration (0.02), and acceptable liver microsomal stability (0.634). The predicted plasma clearance (CL_plasma_ = 8.664 ml/min/kg) indicates moderate clearance, while the half-life (*T*_1/2_ = 0.932 h) classifies it as a short half-life compound. Although the compound shows inhibitory potential towards CYP1A2 (0.983), CYP3A4 (0.791), and CYP2B6 (1.0), no broad CYP promiscuity is predicted, minimising the likelihood of multi-pathway interference.

To further evaluate the safety profile of compound **13a**, the toxicity was predicted using ProTox-III^21^^,^^43^. ProTox-III classifies compounds into six categories according to their predicted median lethal dose (LD_50_), where class one represents the most lethal and class six the safest. The analysis placed compound **13a** in class 4 with a predicted LD_50_ of 1000 mg/kg (Supplementary Figures S1–S48). Collectively, these results indicate that compound **13a** possesses balanced drug-like properties and an acceptable predicted safety margin, with only minor immunotoxicity and respiratory alerts, supporting its potential suitability for further development.

### Cytotoxicity evaluation of compounds 13a and XV against CRC cells

**Figure 5.**
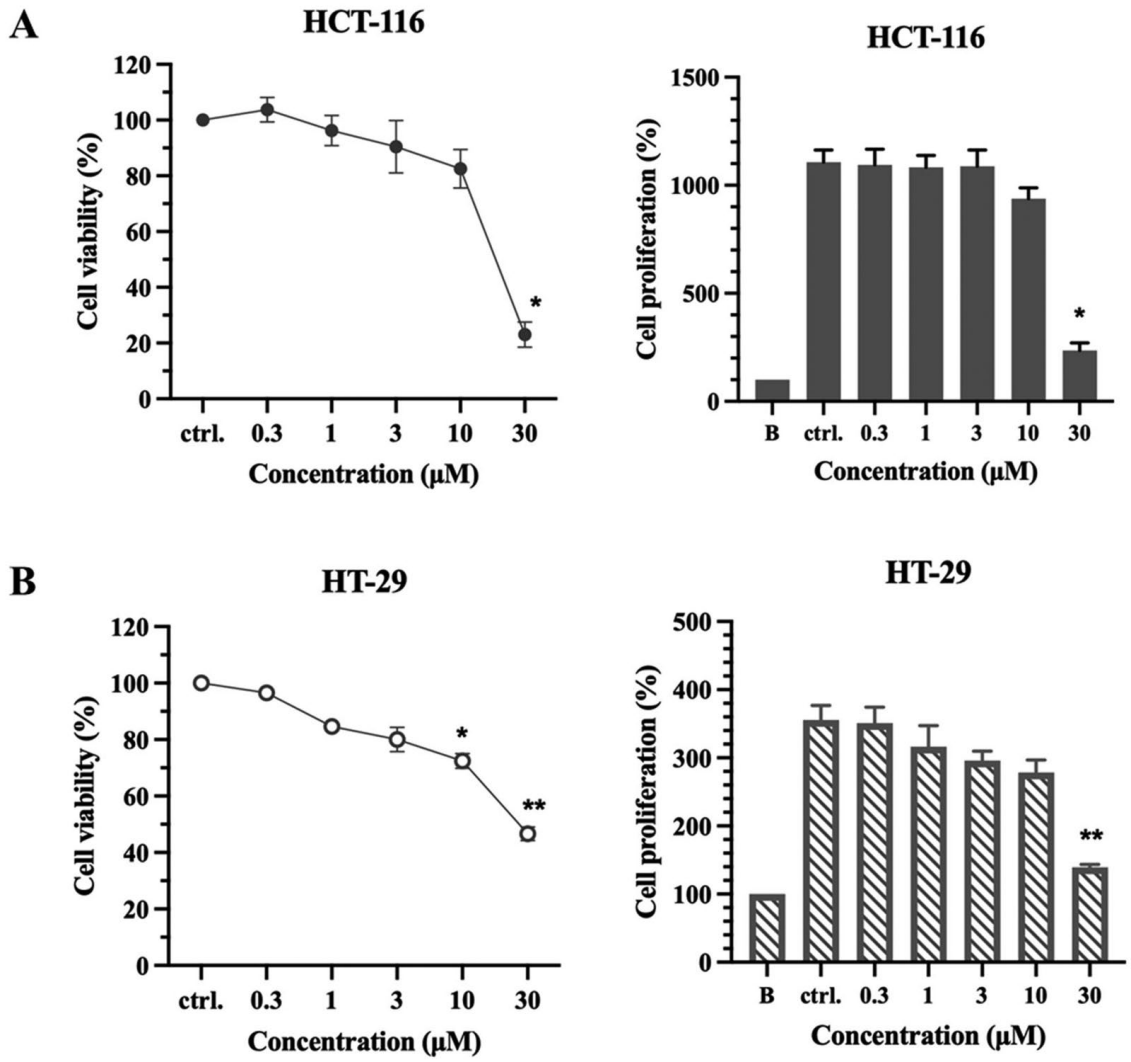
The anticancer effect of LIMK1 inhibitors. Cancer cells were treated with compound **13a** at indicated concentrations (0.3, 1, 3, 10, and 30 μM) for 72 h in (A) HCT-116 and (B) HT-29 cells. The cell viability and cell proliferation were detected by MTT and SRB assays, respectively. All results present mean ± SD of three independent experiments. *p < 0.05 and **p < 0.01 compared to control group (ctrl.).

LIMK1 is overexpressed and showed higher activity in CRC^20^. LIMK1 is related to cancer cell proliferation, migration, invasion, and metastasis. It has been proved that overexpressed LIMK1 in CRC promotes tumour growth and progression, while downregulated LIMK1 inhibits the growth of CRC cells *in vitro* and *in vivo*^20^. Among these compounds, compounds **13a** and **XV** showed the most effective LIMK1 inhibition. To understand the effect of compounds **13a** and **XV** in CRC cell growth, compounds **13a** and **XV** were further evaluated using MTT assay and SRB assay (Table 4). MTT assay is used to measure the cell metabolic activity, while SRB assay is used to assess the protein content. Both assays can be used to evaluate the effects of compounds on cell viability and proliferation^44^. The results indicated that compound **13a** inhibited the metabolic activity of both HCT-116 and HT-29 cells, exhibiting IC_50_ values of 18.24 and 28.78 µM, respectively, as determined by the MTT assay (Figure 5). Additionally, the SRB assay showed that compound **13a** suppressed the growth of HCT-116 and HT-29 cells, with GI_50_ values of 14.65 and 10.36 µM, respectively. In contrast, compound **XV** did not inhibit the growth of HCT-116 cells at a concentration of 30 µM and showed reduced potency in HT-29 cells. These results suggest that compound **13a** possesses a potent anti-proliferative effect on HCT-116 and HT-29 CRC cells.

**Table 4.** The effect of LIMK1 inhibitors in CRC cells.

Compounds	HCT-116		HT-29	
	IC_50_ (μM)	GI_50_ (μM)	IC_50_ (μM)	GI_50_ (μM)
**13a**	18.24 ± 0.9	14.65 ± 0.8	28.78 ± 0.4	10.36 ± 0.6
**XV**	> 30	> 30	> 30	28.77 ± 1.8

### Cell cycle distribution of compound 13a

**Figure 6.**
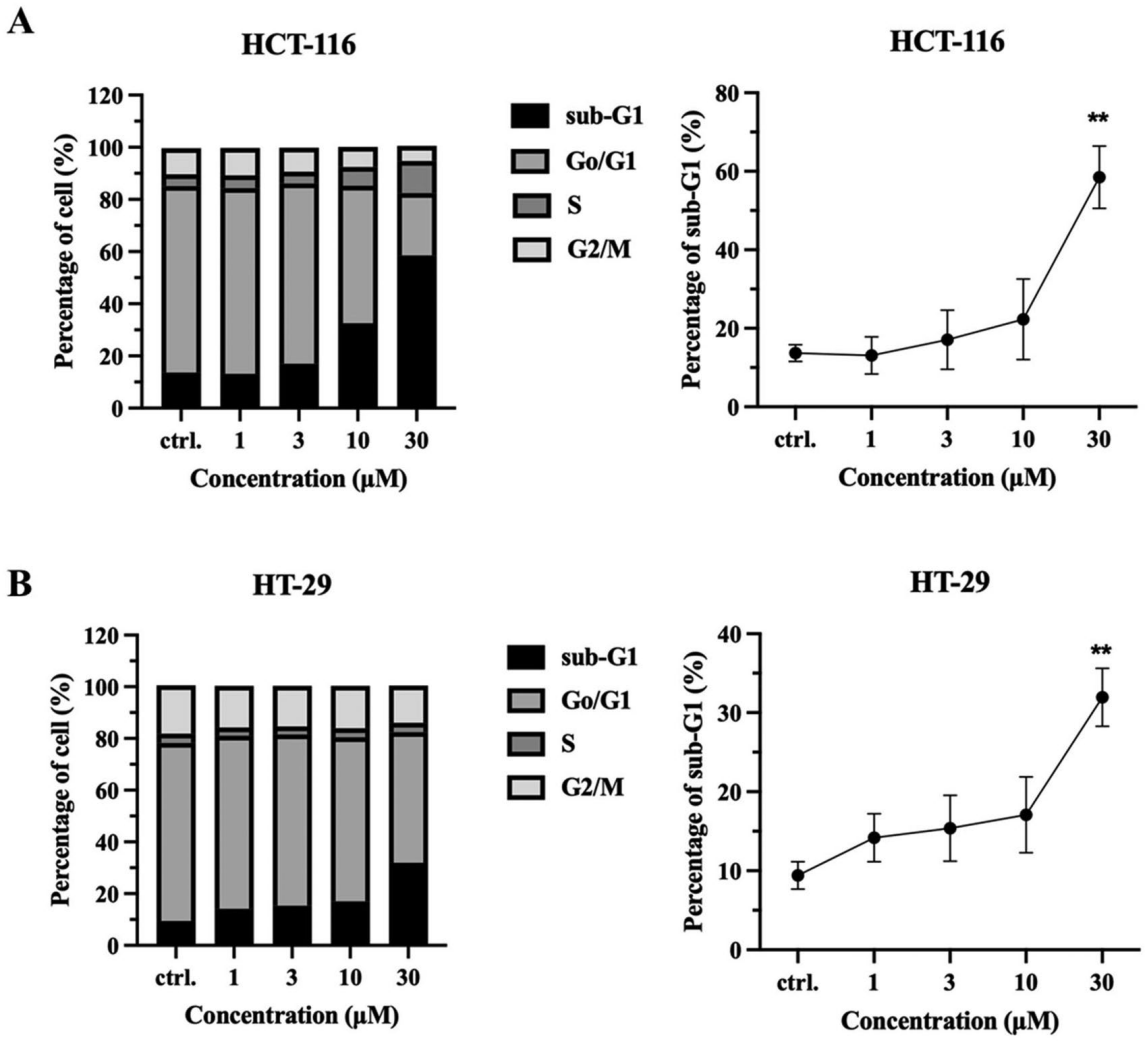
Compound **13a** induced cancer cell apoptosis. (A) HCT-116 and (B) HT-29 cells were treated with compound **13a** with 1, 3, 10, and 30 μM for 72 h. The quantitative analysis of cell cycle distribution and percentage of sub-G1 population were analysed by flow cytometry. **p < 0.01 compared to control group (ctrl.).

To reveal the effect of compound **13a** treatment on the cell cycle, the cell cycle distribution of HCT116 and HT-29 cells was determined after treatment with various concentrations of compound **13a** for 72 h. The results for HCT116 cells indicated a dose-dependent increase in the S phase and a decrease in the G0/G1 phase (Figure 6). Previous research indicated that LIMK1 expression in prostatic hyperplasia cells caused a transient G1/S phase arrest and delayed G2/M progression^45^. Thus, the effects of compound **13a** on the cell cycle might be attributed to its inhibition of LIMK1. In both cell lines, the population of Sub-G1 cells increased in a dose-dependent manner following treatment with compound **13a**, which suggests that compound **13a** induced apoptosis. Accordingly, this result supported that compound **13a** showed anti-proliferative and cytotoxic effects due to LIMK1 inhibition.

## Conclusion

In conclusion, we have designed and synthesised a novel series of compounds featuring a diarylheptanoid scaffold and conducted an extensive exploration of the LIMK1 inhibitory activity to investigate the SAR. Compound **XV** showed the best LIMK1 inhibitory activity. Meanwhile, compound **13a** demonstrated greater cytotoxicity in both HCT-116 and HT-29 cells than compound **XV**. Treatment with compound **13a** in HCT-116 cells led to an increase in the S phase and a decrease in the G0/G1 phase in a dose-dependent manner, like previous LIMK1 inhibitors. These results suggest that compound **13a** has potent and selective LIMK1 inhibitory activity and induces apoptosis in HCT-116 and HT-29 cells, making it a promising lead compound for further optimisation.

## Supplementary Material

Supplementaory material 20251022.docx

## Data Availability

The co-crystal structure of LIMK1 (PDB ID: 3S95) used for molecular docking analysis was downloaded from the Protein Data Bank. Molecular docking was conducted using the software LeadIT. Additional information related to this report can be found online at the following GitHub repository (https://reurl.cc/9D9VGd). The docking poses of the LIMK1 (PDB ID: 3S95) and ligands (compounds **I**, **III**–**VI**, **7c**, **13a**, **XV**, and curcumin) are available in .pdb and .sdf files formats, respectively, on the referenced repository. The docking poses of compounds were visualised using PyMol.
