# Supplementary material for: Exploring diarylheptanoid derivatives to target LIMK1 as potential agents against colorectal cancer
Source: J Enzyme Inhib Med Chem. 2025 Nov 17;40(1):2583826. doi: 10.1080/14756366.2025.2583826 (PMC12624955; doi:10.1080/14756366.2025.2583826)
Supplement: Supplementaory material 20251022.docx [file IENZ_A_2583826_SM9215.docx]

**Exploring Diarylheptanoid Derivatives to Target LIMK1 as Potential Agents Against Colorectal Cancer**

**Supplementary material**

Figure S1. ^1^H NMR (Acetone-*d*­_6_, 300 MHz) spectrum of compound **5a**

Figure S2. ^1^H NMR (MeOD-*d*_4_, 300 MHz) spectrum of compound **5b**

Figure S3. ^1^H NMR (Acetone-*d*_6_, 300 MHz) spectrum of compound **5c**

Figure S4. ^1^H NMR (Acetone-*d*_6_, 300 MHz) spectrum of compound **6a**

Figure S5. ^1^H NMR (MeOD-*d*_4_, 300 MHz) spectrum of compound **6b**

Figure S6. ^1^H NMR (Acetone-*d*_6_, 300 MHz) spectrum of compound **6c**

Figure S7. ^1^H NMR (Acetone-*d*_6_, 300 MHz) spectrum of compound **7a**

Figure S8. ^1^H NMR (MeOD-*d*_4_, 300 MHz) spectrum of compound **7b**

Figure S9. ^1^H NMR (Acetone-*d*_6_, 300 MHz) spectrum of compound **7c**

Figure S10. ^1^H NMR (Acetone-*d*_6_, 300 MHz) spectrum of compound **8a**

 Figure S11. ^1^H NMR (MeOD-*d*_4_, 300 MHz) spectrum of compound **8b**

 Figure S12. ^1^H NMR (Acetone-*d*_6_, 300 MHz) spectrum of compound **9a**

 Figure S13. ^1^H NMR (Acetone-*d*_6_, 300 MHz) spectrum of compound **9b**

 Figure S14. ^1^H NMR (DMSO-*d*_6_, 300 MHz) spectrum of compound **9c**

 Figure S15. ^1^H NMR (Acetone-*d*_6_, 500 MHz) spectrum of compound **10a**

Figure S16. ^13^C NMR and DEPT135 (Acetone-*d*_6_, 125 MHz) spectrum of compound **10a**

 Figure S17. ^1^H NMR (Acetone-*d*_6_, 500 MHz) spectrum of compound **10b**

 Figure S18. ^13^C NMR and DEPT135 (Acetone-*d*_6_, 125 MHz) spectrum of compound **10b**

 Figure S19. ^1^H NMR (Acetone-*d*_6_, 500 MHz) spectrum of compound **10c**

Figure S20. ^13^C NMR and DEPT135 (Acetone-*d*_6_, 125 MHz) spectrum of compound **10c**

 Figure S21. ^1^H NMR (Acetone-*d*_6_, 500 MHz) spectrum of compound **11a**

 Figure S22. ^13^C NMR and DEPT135 (Acetone-*d*_6_, 125 MHz) spectrum of compound **11a**

 Figure S23. ^1^H NMR (Acetone-*d*_6_, 500 MHz) spectrum of compound **11b**

 Figure S24. ^13^C NMR and DEPT135 (Acetone-*d*_6_, 125 MHz) spectrum of compound **11b**

 Figure S25. ^1^H NMR (Acetone-*d*_6_, 500 MHz) spectrum of compound **11c**

 Figure S26. ^13^C NMR and DEPT135 (Acetone-*d*_6_, 125 MHz) spectrum of compound **11c**

 Figure S27. ^1^H NMR (CDCl_3_, 300 MHz) spectrum of compound **13a**

 Figure S28. ^1^H NMR (CDCl_3_, 300 MHz) spectrum of compound **13b**

Figure S29. ^1^H NMR (CDCl_3_, 300 MHz) spectrum of compound **14a**

 Figure S30. ^1^H NMR (CDCl_3_, 300 MHz) spectrum of compound **14b**

 Figure S31. ^1^H NMR (CDCl_3_, 300 MHz) spectrum of compound **15a**

 Figure S32. ^1^H NMR (CDCl_3_, 300 MHz) spectrum of compound **15b**

 Figure S33. ^1^H NMR (Acetone-*d*_6_, 300 MHz) spectrum of compound **16a**

Figure S34. ^13^C NMR and DEPT135 (Acetone-*d*_6_, 125 MHz) spectrum of compound **16a**

 Figure S35. ^1^H NMR (Acetone-*d*_6_, 300 MHz) spectrum of compound **16b**

 Figure S36. ^13^C NMR and DEPT135 (Acetone-*d*_6_, 125 MHz) spectrum of compound **16b**

 Figure S37. ^1^H NMR (Acetone-*d*_6_, 300 MHz) spectrum of compound **17a**

 Figure S38. ^13^C NMR and DEPT135 (Acetone-*d*_6_, 125 MHz) spectrum of compound **17a**

 Figure S39. ^1^H NMR (Acetone-*d*_6_, 300 MHz) spectrum of compound **17b**

 Figure S40. ^13^C NMR and DEPT135 (Acetone-*d*_6_, 125 MHz) spectrum of compound **17b**

 Figure S41. ^1^H NMR (Acetone-*d*_6_, 300 MHz) spectrum of compound **18**

 Figure S42. ^13^C NMR and DEPT135 (Acetone-*d*_6_, 125 MHz) spectrum of compound **18**

 Figure S43. ^1^H NMR (CDCl_3_, 300 MHz) spectrum of compound **20**

Figure S44. ^1^H NMR (DMSO-*d*_6_, 300 MHz) spectrum of compound **21**

 Figure S45. ^13^C NMR and DEPT135 (Acetone-*d*_6_, 125 MHz) spectrum of compound **21**


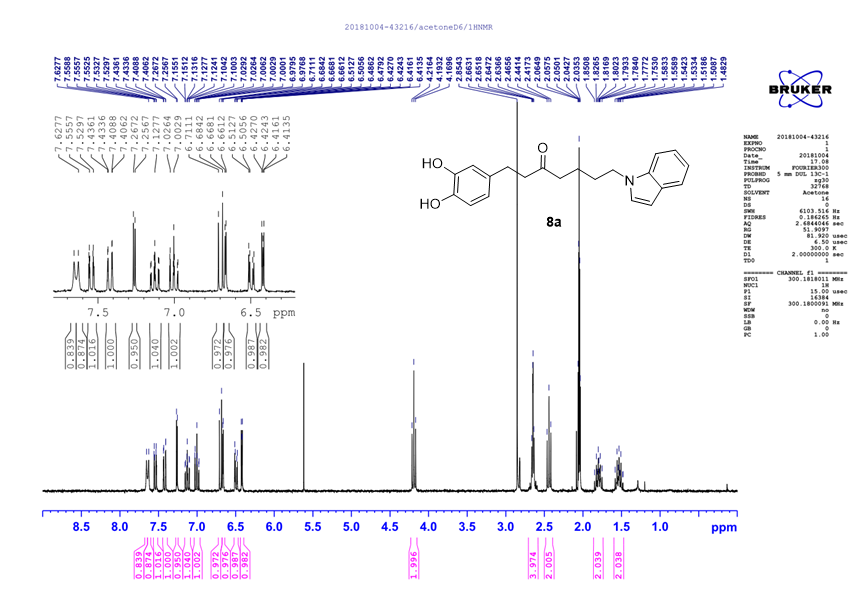


Figure S46. ^1^H NMR (Acetone-*d*_6_, 300 MHz) spectrum of compound **22**


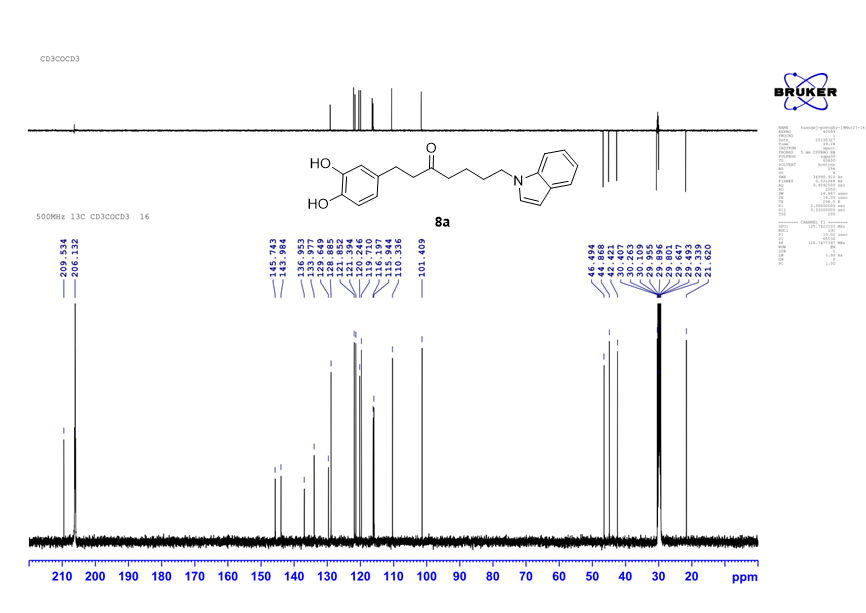


Figure S47. ^13^C NMR and DEPT135 (Acetone-*d*_6_, 125 MHz) spectrum of compound **22**


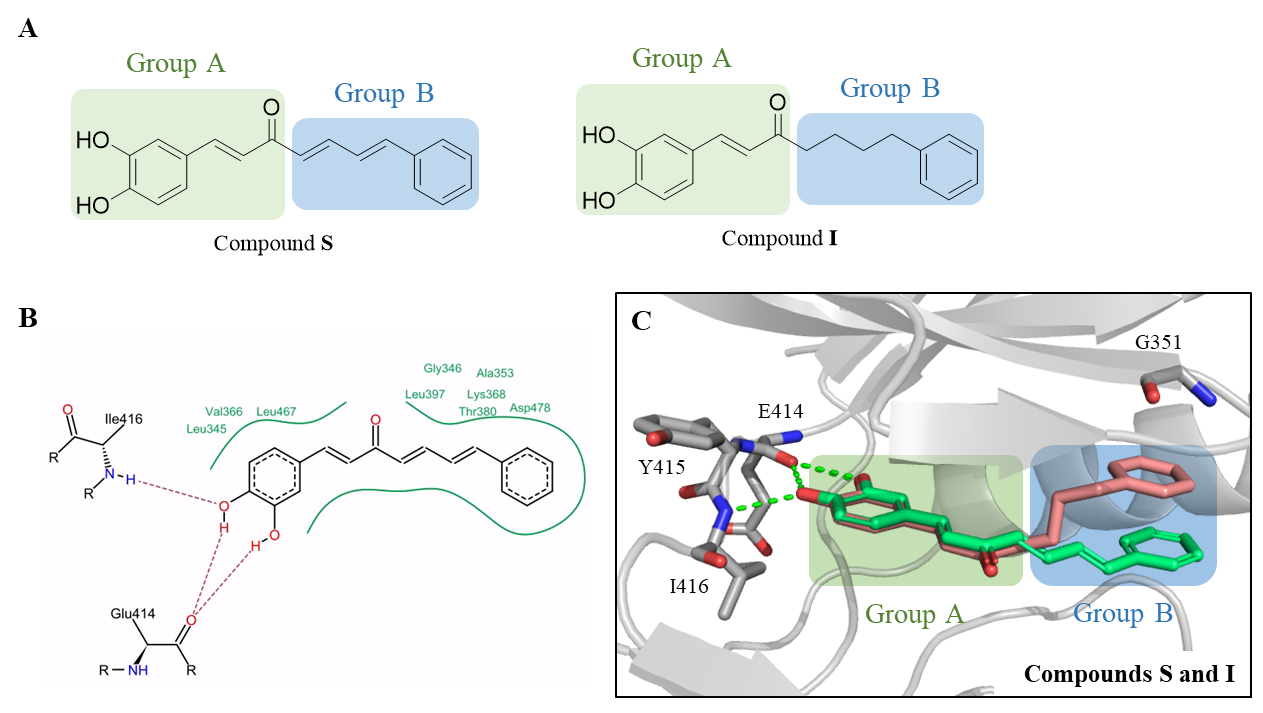


Figure S48. Interaction analysis between compounds **S** and **I**. (A) Chemical structure of compounds **S** and **I**. Each structure is divided into two groups: Group A (caffeoyl group), which is common to both compounds, and Group **B**, which differs between the two (an unsaturated chain in compound **S** versus a saturated chain in compound **I**) (B) Two-dimensional interaction representation generated by LeadIT. Dashed lines indicate hydrogen bonds. Solid green lines indicate hydrophobic interactions. (C) Superimposed docking poses of compounds **S** (salmon) and **I** (apple-green).


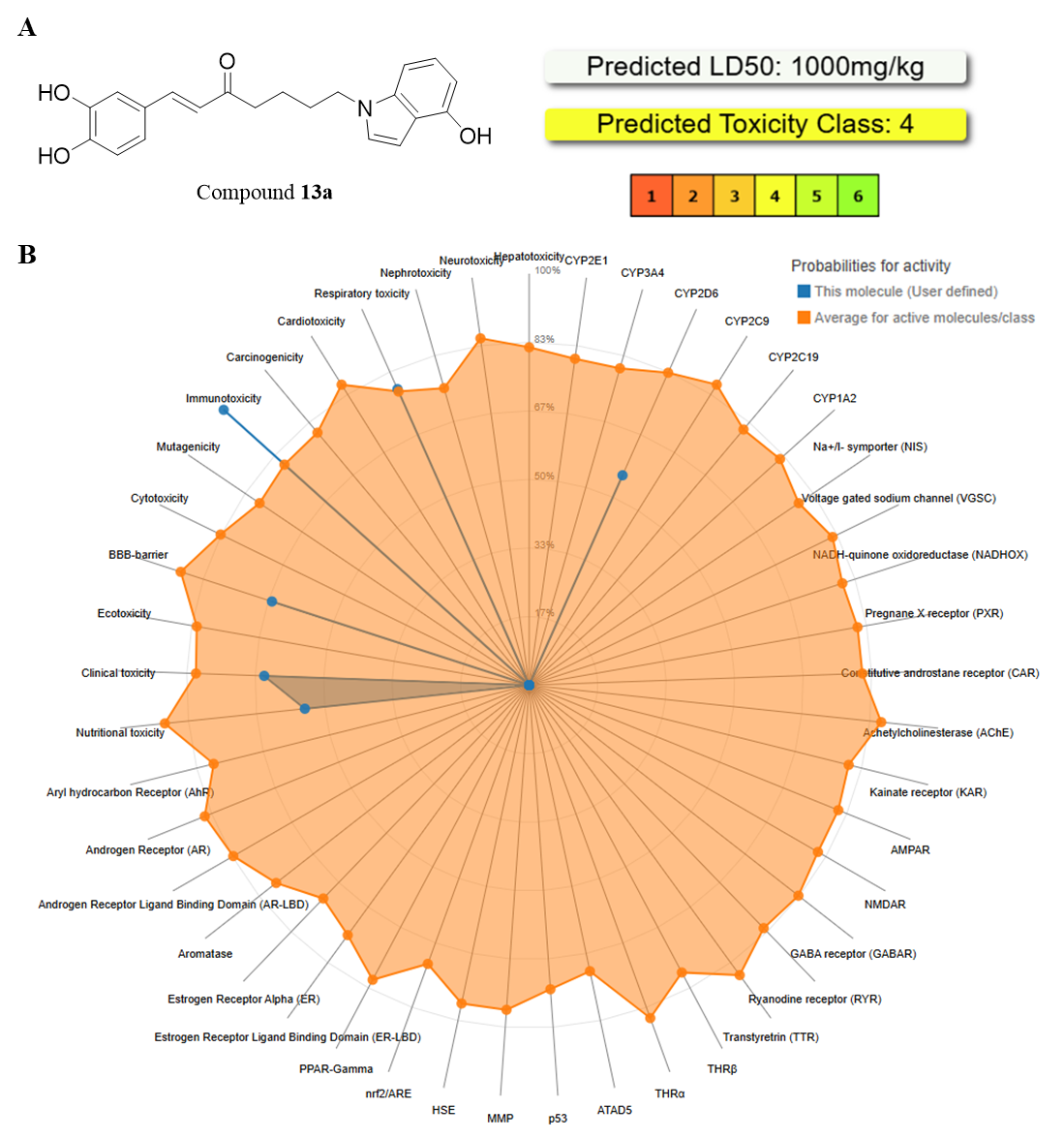


Figure S49. Predicted toxicity of compound **13a**. (A) Chemical structure of compound **13a** and predicted LD₅₀ value of 1000 mg/kg and assignment to toxicity class 4. (B) Radar plot showing predicted probabilities for various toxicological endpoints and off-target activities. The blue dots represent the predictions for compound **13a**, while the orange area indicates the average values for active molecules within each class. Compound **13a** exhibited overall low predicted risks, with only modest signals in immunotoxicity and respiratory toxicity.

**Supplementary Table S1.** The selected kinases in this study and corresponding assays.

| **Z′-LYTE Kinase Assay** | | | |  | | **LanthaScreen Eu Kinase Binding Assay** | |  | | **Adapta Universal Kinase Assay** | |
| --- | --- | --- | --- | --- | --- | --- | --- | --- | --- | --- | --- |
| AKT1  (PKB alpha) | ERBB4  (HER4) | MINK1 | PRKACA  (PKA) |  | BRAF | |  | | DAPK1 | |  |
| AURKA  (Aurora A) | FGFR1 | MKNK1  (MNK1) | PRKCA  (PKC alpha) |  | EPHA3 | |  | |  | |  |
| BTK | HIPK1 (Myak) | NEK1 | ROCK1 |  | ICK | |  | |  | |  |
| CAMK2B  (CaMKII beta) | IKBKB  (IKK beta) | NTRK1  (TRKA) | RPS6KA3  (RSK2) |  | LIMK1 | |  | |  | |  |
| CDK2/cyclin A | IRAK4 | PAK2  (PAK65) | SRC |  | LIMK2 | |  | |  | |  |
| CSNK1D  (CK1 delta) | LCK | PAK2  (PAK65) | STK3  (MST2) |  | MAP2K1  (MEK1) | |  | |  | |  |
| CSNK1G1  (CK1 gamma 1) | MAP3K9  (MLK1) | PAK4 |  |  | TGFBR1  (ALK5) | |  | |  | |  |
| DCAMKL1  (DCLK1) | MAPK3  (ERK1) | PIM1 |  |  | VRK2 | |  | |  | |  |
| DYRK1B | MARK3 | PLK2 |  |  | WEE1 | |  | |  | |  |

**Supplementary Table S2.** Predicted physicochemical property, medicinal chemistry rules, absorption, distribution, metabolism, and excretion (ADME) of compound **13a**.

| **Physicochemical Property** | | |
| --- | --- | --- |
| Property | Value | Comment |
| Molecular Weight | 351.15 | Contain hydrogen atoms.  Optimal:100~600 |
| logP | 2.954 | The logarithm of the n-octanol/water distribution coefficients at pH = 7.4. |
| TPSA | 82.69 | Topological Polar Surface Area.  Optimal:0~140 |
| **Medicinal Chemistry Rules** | | |
| Property | Value | Comment |
| Lipinski Rule | 0.0 | MW ≤ 500; logP ≤ 5; Hacc ≤ 10; Hdon ≤ 5  If two properties are out of range, a poor absorption or permeability is possible; one is acceptable |
| Pfizer Rule | 0.0 | logP > 3; TPSA < 75  Compounds with a high log P (> 3) and low  TPSA (< 75) are likely to be toxic. |
| GSK Rule | 0.0 | MW ≤ 400; logP ≤ 4  Compounds satisfying the GSK rule may have a more favorable ADMET profile |
| Golden Triangle | 0.0 | 200 ≤ MW ≤ 500; -2 ≤ logD ≤ 5  Compounds satisfying the Golden Triangle rule may have a more favorable ADMET profile |
| **Absorption** | | |
| Property | Value | Comment |
| Caco-2 Permeability | -5.091 | Optimal: higher than -5.15 Log unit |
| P-glycoprotein substrate | 0.02 | Category 1: substrate  Category 0: Non-substrate;  The output value is the probability of being a P-glycoprotein substrate |
| Human Intestinal Absorption (HIA) | 0.038 | Category 1: HIA+( HIA < 30%);  Category 0: HIA-( HIA ≥ 30%);  The output value is the probability of being HIA+ |
| **Distribution** | | |
| Property | Value | Comment |
| Plasma Protein Binding | 95.385 | Optimal: < 90%.  Drugs with high protein-bound may have a low therapeutic index. |
| Blood-Brain Barrier Penetration | 0.02 | Category 1: BBB+  Category 0: BBB-  The output value is the probability of being BBB+ |
| **Metabolism** | | |
| Property | Value | Comment |
| Human liver microsomal (HLM) stability | 0.634 | Category 1: unstable (HLM ≤ 30 min)  Category 0: stable (HLM > 30 min)  The output value represents the probability of human liver microsomal instability, with values closer to 1 indicating a higher likelihood of instability. The range is between 0 and 1. |
| CYP1A2 inhibitor | 0.983 | Category 1: Inhibitor  Category 0: Non-inhibitor  The output value is the probability of being an inhibitor. |
| CYP2C19 inhibitor | 0.242 |  |
| CYP2C9 inhibitor | 0.035 |  |
| CYP2D6 inhibitor | 0.006 |  |
| CYP3A4 inhibitor | 0.791 |  |
| CYP2B6 inhibitor | 1.0 |  |
| CYP1A2 substrate | 0.028 | Category 1: Substrate  Category 0: Non-substrate  The output value is the probability of being a substrate. |
| CYP2C19 substrate | 0.0 |  |
| CYP2C9 substrate | 0.237 |  |
| CYP2D6 substrate | 0.784 |  |
| CYP3A4 substrate | 0.0 |  |
| CYP2B6 substrate | 0.0 |  |
| **Excretion** | | |
| Property | Value | Comment |
| CL_plasma_ | 8.664 | The unit of predicted CL_plasma_ penetration is ml/min/kg. |
| T_1/2_ | 0.932 | The unit of predicted T_1/2_ is hours. |
